# Permanent facial palsy and recurrence rate after surgery for benign parotid tumors: pairwise and network meta-analysis of different parotid surgery techniques

**DOI:** 10.3389/fsurg.2026.1836835

**Published:** 2026-05-07

**Authors:** Caroline Bernhard, Peter Schlattmann, Orlando Guntinas-Lichius

**Affiliations:** 1 Department of Otorhinolaryngology, Jena University Hospital, Jena, Germany; 2 Department of Medical Statistics, Computer Sciences and Data Sciences, Jena University Hospital, Jena, Germany; 3 Facial-Nerve-Center, Jena University Hospital, Jena, Germany

**Keywords:** complications, extracapsular dissection, facial paralysis, network meta-analysis, parotidectomy, surgery, tumor recurrence

## Abstract

**Background:**

Parotid surgery for benign tumors has shifted from total parotidectomy (TP) toward less invasive procedures such as partial parotidectomy (PP) and extracapsular dissection (ECD). This study compared these techniques regarding tumor recurrence rate (TRR) and permanent facial palsy rate (FPR).

**Methods:**

A systematic review, pairwise and network meta-analysis were performed on studies reporting TRR and FPR for TP, PP, and ECD. Eligible publications were identified in PubMed, Web of Science, and Cochrane Library through 2024. Rates were analyzed using arcsine transformation and arcsine differences (ASD) with random- or fixed-effects models.

**Results:**

Of 1,249 detected publications, 23 studies with 4,674 adult patients were included. Mean follow-up was 66.0 ± 56.5 months. The average TRR was 2.9 ± 2.9%, and the average FPR was 3.0 ± 5.5%. Network meta-analysis showed no significant TRR difference for ECD (ASD: −0.00; 95% CI: −0.06–0.06) or TP (ASD: −0.07; 95% CI: −0.17–0.04) vs. PP in the random-effects model. For FPR, no significant differences were found between TP and PP or between ECD and PP. Only one study compared ECD with TP for FPR, preventing a meta-analysis.

**Conclusions:**

The findings suggest that, when feasible, ECD appears to offer recurrence risks similar to PP, while both may have slightly higher TRR than TP. Permanent facial palsy risk seems comparable between ECD and PP and may be lower for ECD/PP than for TP. The results should be interpreted with caution, as there is a lack of studies with long-term follow-up. Well-designed randomized surgical studies with long-term follow-up are needed.

## Introduction

Surgery of parotid tumors started in the nineteenth century with tumor enucleation. Due to the high recurrence rates (1), tumor enucleation was abandoned when total parotidectomy (TP) as a more formal surgical techniques with exposure of the facial nerve was established in the mid-twentieth century (2). With the aim for more minimal-invasive surgery, especially to reduce the risk of facial palsy without increasing the risk of tumor recurrence, less extended partial parotidectomy (PP) techniques were developed (3–6). Whereas any parotidectomy technique is guided by a facial nerve dissection, the latest development, extracapsular dissection (ECD) approaches the tumor directly without facial nerve dissection (7). Extracapsular dissection is tumor-guided surgery. In contrast to parotidectomy, extracapsular dissection needs a patient selection, as this technique is primarily reserved for benign, mobile and superficial tumors. In order to provide a comprehensive classification system for parotid surgery techniques, Quer et al. developed the European Salivary Gland Society (ESGS; nowadays Multidisciplinary Salivary Gland Society, MSGS) allowing a better comparison of the different surgical approaches (8).

Prospective clinical trials in the field of parotid surgery are sparse (9–12). Prospective trial comparing the different surgical techniques are largely lacking. Most knowledge comes from retrospective monocentric studies. In such a situation, meta-analysis provides a more precise estimate of the effect size and increases the generalizability of the results of individual studies (13, 14). In particular, the discussion about the significance of ECD has led to a series of meta-analyses over the years to date (15–21). To our knowledge, only one network meta-analysis allowing the synchronous comparison of more than two surgical techniques was published so far (22). The outcome measures and their definitions in these meta-analyses are highly variable. The definition of a tumor recurrence is clear. As benign tumor recurrence after parotid surgery can occur years later (23), it is important to have a sufficient follow-up time. Concerning complications, permanent facial palsy is the most important sequela. Here, a standardized measure, i.e., using a facial grading system and again, a sufficient follow-up time, is needed.

Therefore, the aim of the present study was to perform meta-analyses, and if feasible, network meta-analyses comparing the recurrence rates and permanent facial palsies rates in clinical trials comparing at two or more different parotid surgery techniques with an adequate follow-up time.

## Material and methods

This study followed the Preferred Reporting Items for Systematic Reviews and Meta-Analyses (PRISMA) guidelines (24). Ethical approval and patient informed consent were not required for a meta-analysis.

### Data sources and literature search

Electronic databases (PubMed/MEDLINE, Web of Science, Cochrane Library) were screened. This was supplemented by manual research. The Medical Subject Heading (MeSH) terms and combinations that were used for the search and the number of hits are listed in Supplementary Table 1. The literature search revealed 1294 results until the end of February 2024.

### Selection of studies

Initially, all hits from the systematic literature search were imported from the literature databases into the literature management program EndNote (version EndNote X9.3.3, Clarivate Analytics, Philadelphia). Two independent reviewers (C.B.; O.G.L.) reviewed abstracts and full texts. If they came to a different conclusion, a joint decision was made in a discussion. All studies were assessed against general exclusion criteria: review articles, duplicate patients, absence of essential data, multiple use of same patient dataset and animal studies. Further exclusion criteria were as follows: non-English or non-German language; full text not available; insufficient reported data or non-extractable data; case series including less than ten patients; subgroup analyses of patients from larger studies; article types including reviews, case reports, conference abstracts, letters to the editor, or book chapters. No restrictions on the publication date were applied, but peer-reviewed journal publication was a requirement for article inclusion.

### Eligibility criteria

**Figure 1:**
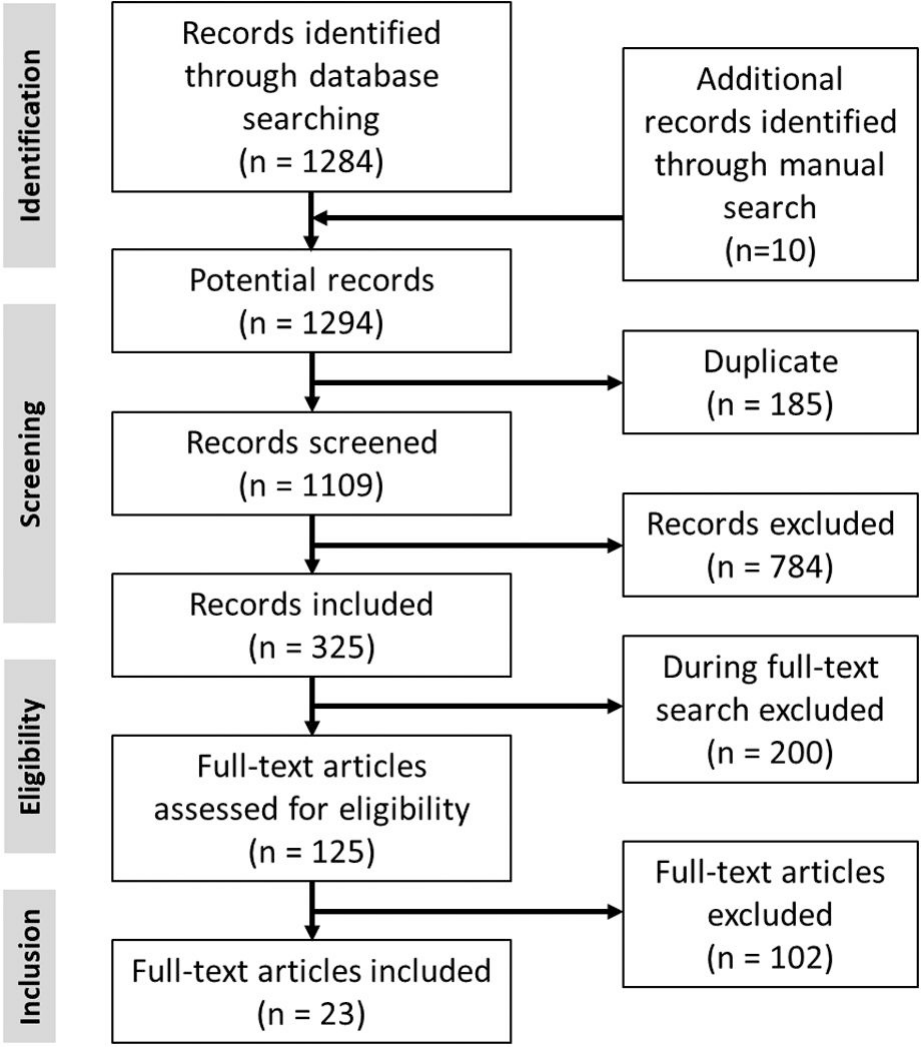
PRISMA chart showing the process of the selection of the included studies.

The PICOS scheme was utilized to establish the eligibility criteria for this study. Patients (P): ≥16 years of age, with benign parotid tumor. Intervention (I): total parotidectomy (TP), partial parotidectomy (PP), and extracapsular dissection (ECD). The techniques were defined in accordance to the European Salivary Gland Society (ESGS) classification (8). That means that studies classifying a parotid technique as superficial parotidectomy, lateral parotidectomy, or partial lateral parotidectomy were assigned to PP. Comparison (C): comparison of these three parotid surgery techniques. Outcomes (O): Two outcomes were analyzed: Rate of permanent facial palsy and rate of tumor recurrence. A permanent facial palsy was defined as a facial palsy persisting for ≥12 months. Furthermore, the facial palsy had to be classified by the House-Brackmann grading (25). Study design (S): Retrospective and prospective cohort studies, case-control studies, case series, and randomized clinical trials (RCTs) were included. Studies were included when at least two of the three parotid techniques (TP, PP, ECD) were compared. The studies needed a follow-up time of ≥12 months. Twenty-three (23) articles were finally included in the analysis (Figure 1). The reasons for excluding full-text studies are summarized in Supplementary Table 2. The literature review was conducted independently by two researchers (C.B. and O.G.L.). Different results were discussed and until a consent was reached.

### Data extraction and quality analysis

The relevant data for the statistical analysis was then extracted from the selected studies. A summary table in Excel [version Excel 16.89, Microsoft, Redmond (USA)] presented the year of publication, the mean follow-up duration in months, the Digital Object Identifier (DOI) and the study type of all included studies. Two further Excel tables were created for the subsequent statistical analysis. Only the number of patients treated and the number of events (recurrence or facial nerve palsy) were transferred to these tables according to a four-field table. One of these Excel tables collected the data for the recurrence endpoint, and the other collected the data for the permanent facial nerve palsy endpoint. This separation was necessary to perform two separate meta-analyses. If information was missing, for example if only the maximum or minimum observation period was given, this data was requested from the first, corresponding, and senior author. However, this was not successful in any case. Therefore, some studies, otherwise fulfilling all other criteria, were not included in the meta-analysis (26–28).

The Newcastle-Ottawa Quality Assessment Scale (NOQAS) for cohort studies (http://www.ohri.ca/programs/clinical_epidemiology/oxford.asp) was used by two reviewers (C.B. and O.G.L.) to assess the overall quality of each included study. There was one non-randomized prospective trial. This study and also all cohort studies were additionally assessed with the ‘Risk Of Bias in Non-randomised Studies of Interventions' (ROBINS-I) tool (https://www.unsw.edu.au/research/ndarc/resources/risk_of_bias_tool; Supplementary Table 3). Two studies were randomized trials. These two trials were assessed using version 2 of the Cochrane risk-of-bias tool for randomized trials (RoB 2; https://methods.cochrane.org/bias/resources/rob-2-revised-cochrane-risk-bias-tool-randomized-trials; Supplementary Table 4). The two reviewers evaluated the implementation of this assessment tool and agreed on a method before their independent assessments of the studies. Disagreements were resolved by discussion.

### Statistics

The Excel tables were formatted for coding with the program R (version 4.3.1, R Foundation for Statistical Computing, Vienna) and converted into a CSV file. The R program and the RStudio interface (version 2023.12.0 + 369, Posit, Boston) were used for all calculations in this meta-analysis.

A network meta-analysis allows comparisons of ≥3 treatment arms with each other (29, 30). This made it possible to compare all three surgical methods with each other, even if there was no direct comparison in the primary study. If sufficient studies were available, the aim was to ultimately assess which surgical method was the best in terms of the respective endpoint. For this purpose, a summarized intervention effect was first calculated for each study. In the second step, a summarized estimate of the intervention effects was created from the combination of these partial analyses as a weighted average of the individual studies (31). The primary studies were not considered equally, but weighted differently according to their relevance. This meant that larger studies were weighted more heavily in the analysis than smaller, less relevant studies. This weighting was also reflected in the presentation of the results using a forest plot (32).

Rates were transformed using the arcsine transformation due to sparse or zero events. As a measure of association, we used the arcsine difference (ASD). In forest plots, the effects of the individual studies and the pooled effect of all studies [ASD with a 95% Clopper-Pearson confidence interval (CI)] were presented graphically. Again, ASD was used because in some parotid surgery comparisons no event occurred in the observed groups. This would have meant that zero would have had to be divided by zero when calculating the effect, which would not have yielded an analyzable result. ASD allows a network meta-analysis in such cases (33). Assessment of statistical heterogeneity and inconsistency was performed using Cochran's Q-test. Statistical heterogeneity is quantified as the I-squared measure which represents the variability of the results due heterogeneity between studies or inconsistency. Publication bias was assessed via Egger's test for funnel plot asymmetry. I^2^ statistics were used to quantify statistical heterogeneity (34).

The Risk of Bias in Network Meta-Analysis (RoB-NMA) tool was used to assess the risk of bias in the network meta-analysis (35). This tool comprises three domains, each with three to five statements. The statements can be answered with “true,” “probably true,” “probably false,” “false,” or “no information.” Answers marked “true” indicate a low risk of bias, while negative statements may indicate possible bias in the results or conclusions of the network meta-analysis. For the final assessment, the RoB-NMA tool was combined with the Risk of Bias in Systematic Reviews (ROBIS) tool (36). The ROBIS tool is used to assess the risk of bias in systematic reviews and also comprises three domains, each with three to five key questions. Based on these questions and statements, the overall risk of bias in the analysis was determined.

## Results

### Included studies

An overview of the 23 included studies with a total of 4,674 patients is presented in Table 1. Only three prospective studies could be included. Twenty studies had a retrospective design (observational studies). Some studies included only patients with pleomorphic adenomas and/or Warthin tumors. Other studies included all types of benign parotid tumors. ECD (*n* = 2058), PP (*n* = 2079), and TP (*n* = 537) were represented in 21, 22, and 6 studies, respectively. Two studies compared all three methods. Further three studies compared PP and TP. One study compared ECD and TP. The remaining eighteen studies compared ECD and TP. Twenty studies reported recurrences rates. Five studies reported FPR. The average follow-up time of all studies was 66.0 ± 56.5 months. The follow-up time of the studies reporting TRR was 61.0 ± 42.27 months. The average TRR over all studies was 2.9 ± 2.9% (ECD: 3.2 ± 3.5; PP: 2.8 ± 2.3; TP: 2.0 ± 4.0). The follow-up time of the studies reporting FPR was 40.5 ± 19.6 months. The average overall FPR was 3.0 ± 5.5% (ECD: 1.0 ± 1.1; PP: 2.0 ± 2.2; TP: 19.6).

**Table 1:** List of the included studies.

No.	Authors	Year	Study design	Patients’ histologies (P)	Inter-vention (I)	Control (C)	No. of patients (I/C)	Outcome measures (O)	NOQAS	ROBINS-I
1	Abdwahed et al. (45)	2022	pro, RCT	several	ECD	PP	25/25	TRR	7	NA
2	Barca et al. (46)	2020	retro	PA	ECD	PP	210/51	TRR	7	Moderate
3	Bonavolontà et al. (47)	2019	retro	PA	ECD	PP	194/89	TRR	5	Moderate
4	Cheng et al. (48)	2020	retro	several	ECD	PP	64/80	TRR, FPR	6	Moderate
5	Committeri et al. (49)	2023	retro	several	ECD	PP	356/198	TRR	4	Serious
6	Cristofaro et al. (50)	2014	retro	PA	ECD	PP	153/45	TRR	7	Moderate
7	Dell’Aversana Orabona et al. (51)	2013	retro	several	ECD	PP	176/56	TRR	5	Moderate
8	Guntinas-Lichius et al. (52)	2004	retro	PA	TP	PP	42/253	TRR	4	Moderate
9	Iro et al. (53)	2013	retro	PA	ECD, TP	PP	76/57/86	TRR	7	Low
10	Kadletz et al. (27)	2020	retro	PA, WT	ECD	PP	42/85	TRR	3	Serious
11	Laskaris et al. (54)	2022	retro	several	ECD	PP	123/143	TRR	5	Moderate
12	Laskawi et al. (55)	1996	retro	PA	TP	PP	60/139	TRR	4	Serious
13	Lee et al. (56)	2017	retro	WT	ECD	PP	43/34	TRR, FPR	7	Moderate
14	Mantsopoulos et al. (44)	2018	retro	WT	ECD	TP	96/107	TRR, FPR	4	Moderate
15	Ozturk et al. (57)	2019	retro	several	ECD	PP	58/78	TRR, FPR	5	Moderate
16	Prichard et al. (58)	1992	retro	several	ECD	PP	31/15	TRR	4	Moderate
17	Rafi et al. (59)	2020	pro, RCT	PA, WT	ECD	PP	19/27	TRR	8	NA
18	Riad et al. (60)	2011	pro, non RCT	PA	TP	PP	134/30	TRR	7	Low
19	Schapher et al. (61)	2019	retro	PA	ECD, TP	PP	77/30/31	TRR	5	Moderate
20	Uyar et al. (62)	2011	retro	PA	ECD	PP	21/20	TRR	4	Moderate
21	Vanroose et al. (63)	2023	retro	several	ECD	PP	102/59	TRR	5	Serious
22	Visconti et al. (64)	2022	retro	several	ECD	PP	50/354	TRR	4	Serious
23a	Zheng el al.* (65)	2019	retro	several	ECD	PP	92/189	FPR	4	Moderate
23b	Zheng et al.* (65)	2019	retro	several	ECD	PP	50/99	TRR	5	Moderate

Pro, prospective study; retro, retrospective study; RCT, randomized controlled trial; ECD, extracapsular dissection; PP, partial parotidectomy; TP, total parotidectomy; TRR, tumor recurrence rate; FPR, permanent facial palsy rate; PA, pleomorphic adenoma; WT, Warthin tumor.

* This study allowed a separate analysis of two subcohorts. NOQAS, newcastle-ottawa quality assessment scale; ROBINS-I, risk of bias in non-randomized studies - of interventions; NA, not applicable.

### Risk of bias assessment of the included studies

The NOQAS quality analysis total scores for the retrospective studies varied between 3 and 7 points (cf. Table 1). The NOQAS total score for all studies was good (7-9 points) for 7 studies, moderate (4-6 points) for 16 studies, and low (0–3) for 1 study. The overall bias using the ROBINS-I assessment for all retrospective cohort studies and the one prospective non-randomized trial was low for 2 studies, moderate for 15 studies, and serious for 5 studies (cf. Table 1; details on all domains in Supplementary Table 3). The Risk of Bias Assessment using the Revised Cochrane Risk-of-Bias Tool for Randomized Trials (RoB 2) tool for the two included prospective randomized trials revealed an overall risk of bias with “some concern” (details on all domains in Supplementary Table 4).

### Meta-analysis and network meta-analysis on the rate of recurrence

**Figure 2:**
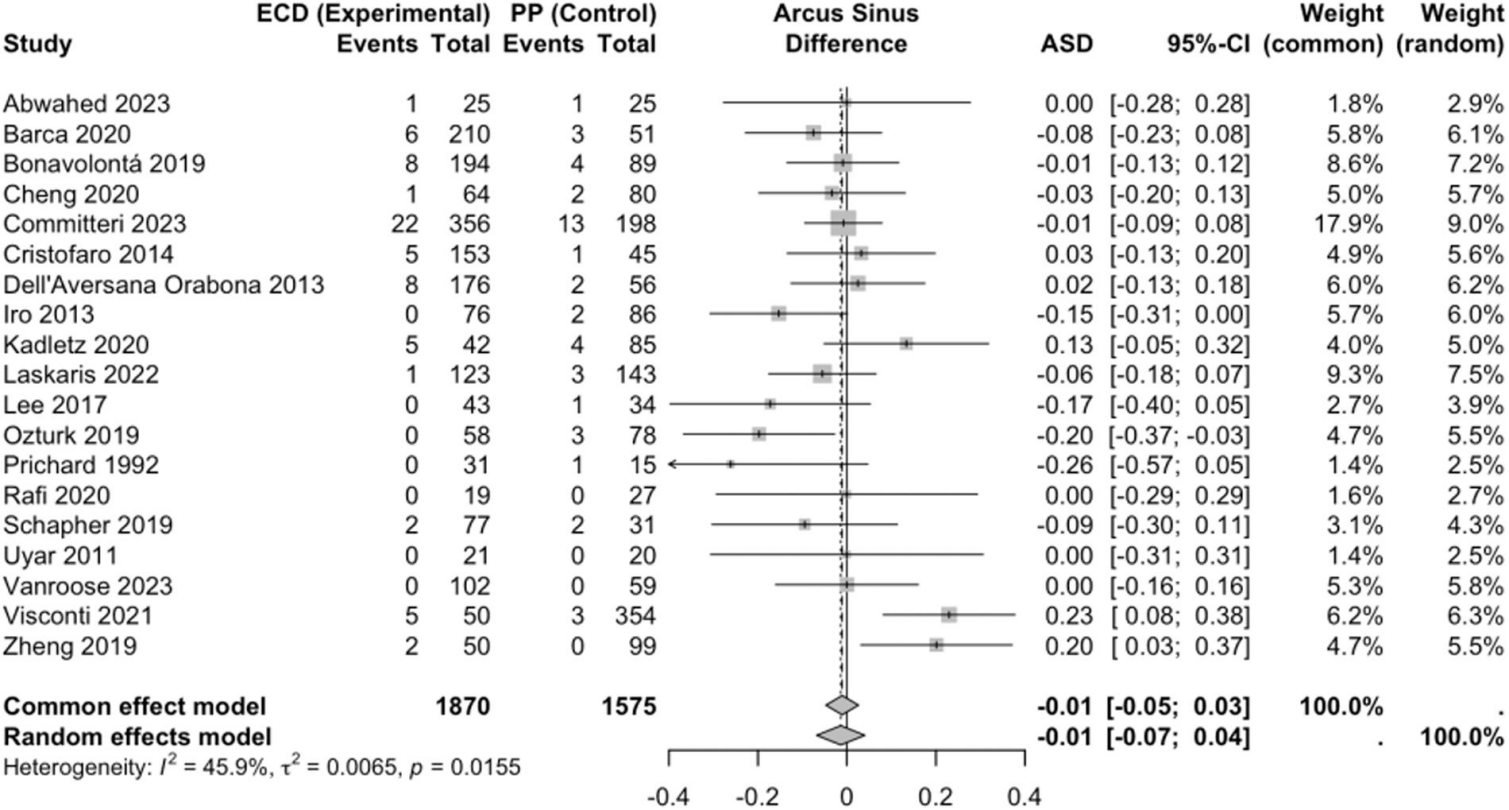
Forest plot for the meta-analysis of ECD versus PP with regard to the endpoint recurrence, showing the results in the model with random and fixed effects. ECD, extracapsular dissection; PP, partial parotidectomy; ASD, effect measure arcsine difference; 95%-CI, 95% confidence interval; ASD <0, fewer recurrences with ECD; ASD >0, more recurrences with ECD.

The comparison between ECD and PP included 19 studies with 3,445 patients (Figure 2). There was no significant difference between both surgical techniques, using a model with random effects (ASD: −0.01; 95%; CI: −0.07 to 0.04) The studies were statistically significant heterogeneous (Chi-square = 33.27, df = 19, *p* = 0.016, I^2^ = 46%).

**Figure 3:**
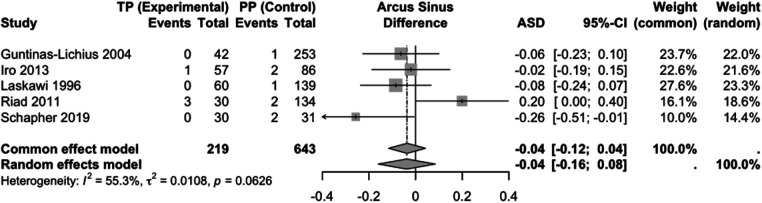
Forest plot for the meta-analysis of TP versus PP with regard to the endpoint recurrence, showing the results in the model with random and fixed effects. TP, total parotidectomy; PP, partial parotidectomy; ASD, effect measure arcsine difference; 95%-CI, 95% confidence interval; ASD <0, fewer recurrences with TP; ASD >0, more recurrences with TP.

Five studies with a total of 862 study participants were included in the comparison of TP vs. PP (Figure 3). There was no statistically significant difference between the two groups in the random-effects model (ASD: −0.04; 95% CI: −0.16 to 0.08). There was some heterogeneity (Chi-square = 8.94, df = 4, *p* = 0.063, I^2^ = 55%).

**Figure 4:**
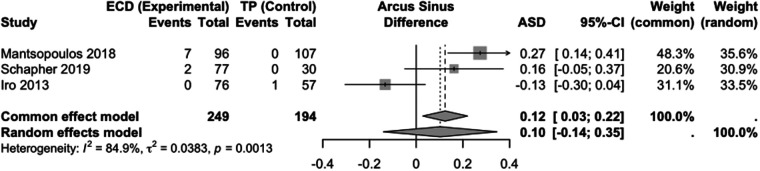
Forest plot for the meta-analysis of ECD versus TP with regard to the endpoint recurrence, showing the results in the model with random and fixed effects. ECD, extracapsular dissection; TP, total parotidectomy; ASD, effect measure arcsine difference; 95%-CI, 95% confidence interval; ASD <0, fewer recurrences with ECD; ASD >0, more recurrences with ECD.

Data from three studies with a total of 443 patients were available for the comparison of ECD and TP (Figure 4). The fixed-effects model showed a significantly higher incidence of recurrence in the experimental ECD group (ASD: 0.10; 95% CI: −0.14 to 0.35) but there was clear heterogeneity (Chi-square =13.24, df = 2, *p* = 0.001, I^2^ = 85%).

**Figure 5:**
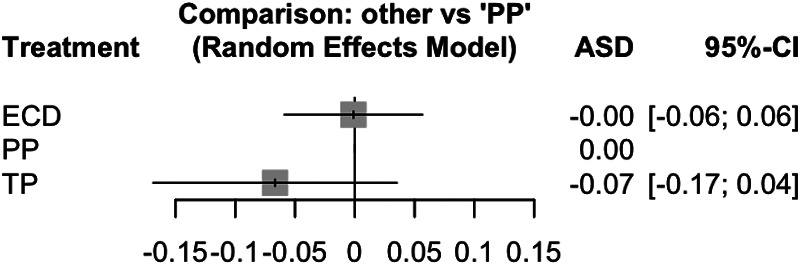
Forest plot for the network meta-analysis of ECD versus PP and TP with regard to the endpoint recurrence with presentation of the result in the model with random effects. ECD, extracapsular dissection (experimental intervention); PP, partial parotidectomy (control intervention); TP, total parotidectomy (experimental intervention); ASD, effect measure arcsine difference; 95%-CI, 95% confidence interval; ASD <0; fewer recurrences with ECD or TP compared to PP; ASD >0; more recurrences with ECD or TP compared to PP.

The network meta-analysis compared the three surgical methods regarding recurrence (Figure 5). Here we find statistically significant inconsistency (Chi-square =13.62, df = 3, *p* = 0.035. Thus, a random effects model was applied. In this model, there was no significant difference for either ECD (ASD: −0.00; 95% CI: −0.06–0.06) or TP (ASD: −0.07; 95% CI: −0.17 to 0.04) compared to PP.

### Meta-analysis and network meta-analysis on the rate of permanent facial palsy

Five included studies investigated the endpoint of permanent facial nerve palsy. This resulted in a total number of 841 patients.

**Figure 6:**
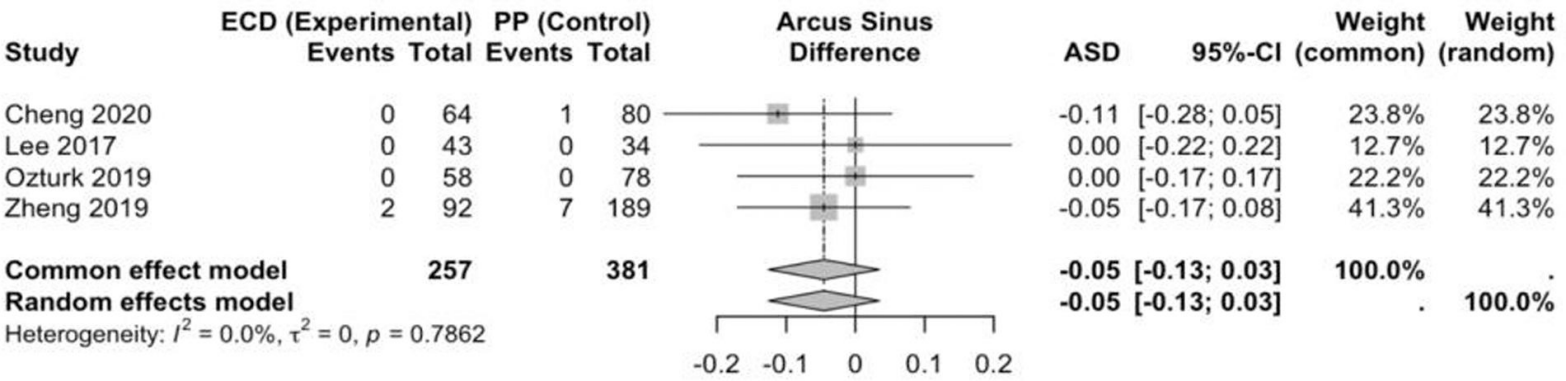
Forest plot for the meta-analysis of ECD versus PP with regard to the endpoint permanent facial nerve palsy with presentation of the results in the model with random and fixed effects. ECD, extracapsular dissection; PP, partial parotidectomy; ASD, effect measure arcsine difference; 95%-CI, 95% confidence interval; ASD <0, fewer permanent facial nerve palsies with ECD; ASD >0, more permanent facial nerve palsies with ECD.

The comparison between ECD and PP was based on 638 patients (Figure 6). The fixed effects model (ASD: −0.05; 95% CI: −0.13 to 0.03 revealed no statistically significant difference between the intervention groups. There was no heterogeneity (Chi-squared = 1.06, df =3, *p* = 0.786, I^2^ = 0%).

**Figure 7:**
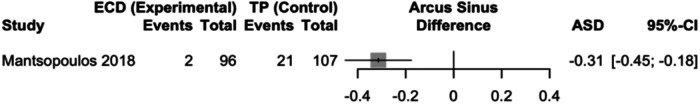
Forest plot for the analysis of ECD versus TP with regard to the endpoint permanent facial nerve palsy. As only one study performed this comparison, a meta-analysis was not possible. ECD, extracapsular dissection; TP, total parotidectomy; ASD, effect measure arcsine difference; 95%-CI, 95% confidence interval; ASD <0, fewer permanent facial nerve palsies with ECD; ASD >0, more permanent facial nerve palsies with ECD.

The comparison between ECD and TP included 203 patients (Figure 7). Only one study was found comparing ECD and TP.

**Figure 8:**
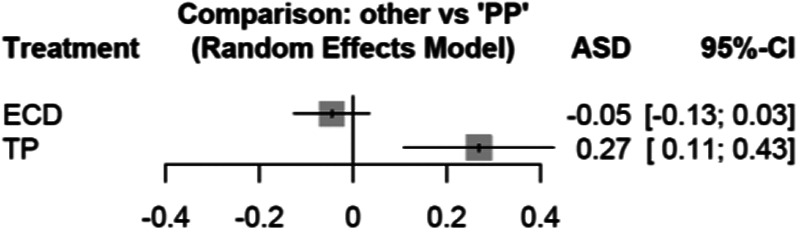
Forest plot for the network meta-analysis of PP versus ECD or versus TP with regard to the endpoint permanent facial palsy. PP, partial parotidectomy; ECD, extracapsular dissection; TP, total parotidectomy; ASD, effect measure arcsine difference; 95%-CI, 95% confidence interval; ASD <0, fewer permanent facial palsies with ECD or TP compared to PP; ASD >0, more permanent facial palsies with ECD or TP compared to PP.

A network meta-analysis did not show inconsistency (Chi-squared = 0, df = 0). The results are shown in Figure 8 with a higher risk for TP vs. PP (95% CI: 0.27, 0.14 to 0.43), but no significant difference between ECD and PP (95% CI: −0.05, −0.03 to 0.03).

### Assessment of publication bias

A funnel plot was created for each pairwise meta-analysis and for the network meta-analysis on the TRR (Supplementary Figure 1A-D). Egger's test did not reveal a significant publication bias for all meta-analyses: ECD vs. PP (*p* = 0.595); TP vs. PP (*p* = 0.841); ECD vs. TP (*p* = 0.724); ECD vs. PP vs. TP (*p* = 0.703). Concerning the endpoint FPR, only an analysis for the comparison ECD vs. PP was feasible (Supplementary Figure 1E). There was also no publication bias for this comparison (*p* = 0.702).

The statements made by the RoB-NMA tool for checking the network meta-analysis for bias could be answered nine times with “true,” once with “probably true,” and three times with “false” (Supplementary Table 5). In one case, no statement could be made due to the data situation. Although most of the statements in the RoB-NMA tool were answered in the affirmative, the three negative statements indicated a certain risk of bias (Supplementary Table 6). The evaluation with the ROBIS tool also yielded predominantly “true” answers. Only the review of the individual studies included for bias had to be rejected. In domain three of the ROBIS tool, this resulted in a certain risk of bias at the level of the systematic review. In summary, the network meta-analysis therefore shows a partial risk of bias in the results. However, a low risk of bias was found for the conclusions drawn.

## Discussion

Theoretically, one would expect that the more extensive the surgery on the parotid gland, the lower the recurrence rate would be and the higher the rate of facial palsy would be. In fact, the results of the presented pairwise and network meta-analyses were not quite so clear. In the random effects model, however, there was no significant difference in either case. Concerning the FPR, ECD showed a lower FPR in the fixed-effects model compared to TP., TP resulted in a significantly higher FPR compared to PP. TP could therefore be the worse surgical method in terms of permanent facial nerve palsy compared to both ECD and PP. ECD and PP did not prove to be better or worse than the other surgical method in either model for the endpoint permanent facial nerve palsy.

Some variability of the TPR and FPR in-between the included studies can be explained with an uncontrolled selection bias due the retrospective or non-randomized design of most of the included studies. Therefore, the results must be interpreted with caution. One has to avoid to compare apples and oranges (37): ECD is typically only performed for 2-4 cm mobile superficial tumors and by experienced surgeons. Alternatives for any tumor in the superficial part of the parotid gland, also for tumors >4 cm, are PP of ESGS level I, level II or both, depending the tumor localization (8). Less experienced surgeons typically start parotid surgery with parotidectomy techniques (38). ECD is reserved for experienced parotid surgery, as the surgeon must be able to switch surgery from ECD to a parotidectomy technique, if the intraoperative situs shows that ECD is not feasible (39). Tumor of the deep lobe are mostly treated by TP. The risk of facial palsy increases with the tumor size and tumor localization (40), hence, especially in cases where an ECD cannot be performed. For a correct comparison of ECD, PP and TP, theoretically only patients who had a mobile, superficial tumor operated by experienced surgeons should be considered. Such a meta-analysis is not possible with the available data.

Another important issue was a clear definition of the extent of surgery. ECD (tumor removal without facial nerve dissection, <1 level resected), PP (resection of ≥ 1 level) and TP (complete removal of the gland, level I-IV) are clearly defined. All other terms have no clear definition (“partial superficial parotidectomy”, “limited superficial parotidectomy”, “subtotal parotidectomy”, “near-total parotidectomy”) or are used by tradition (“superficial parotidectomy”, “lateral parotidectomy”), although the peripheral facial nerve is not dissected completely and the lateral part of the parotid gland is not completely resected in all cases. Hence, for a meta-analysis is seems to be impossible to really differentiate between PP and all other types of incomplete resection of the parotid gland. Therefore, we decided to subsume all these cases as PP. The other difference to prior meta-analyses is (overview in Supplementary Table 7), that we included only studies with a follow-up of ≥12 months and reporting a standardized facial nerve grading. This was important to synthesize reliable data on recurrence rates and permanent facial palsy rates. Since recurrences of benign tumors can also occur years later (23, 41), it would of course have been desirable to only look at studies with even longer follow-up. However, there are not enough of these studies for a meta-analysis. Furthermore, we were originally interested in studies using a modern and more reliable grading system (for instance, Sunnybrook grading or eFACE) than the House-Brackmann grading scale (42, 43). Unfortunately, such facial gradings have not yet been used in parotid surgery studies, so there was no choice but to use the still widely used House-Brackmann grading.

Due to the strict selection criteria, the present analysis shows differences compared to the eight prior meta-analyses published between 2012 and 2023 (Supplementary Table 7). The average TRR was 3.2%, 2.8%, and 2.0% for ECD, PP and TP, respectively. The average overall FPR was 1.0%, 2.0%, and 19.6%, respectively. The later rate for TP seems to be an outlier, as only one study could be included to calculate the FPR after TP (44). The authors themselves admit a selection bias: TP was chosen for multifocal Warthin tumors with diffuse foci or large lesions in widespread contact with the facial nerve, hence, a very special and difficult surgical situation (44). In prior meta-analyses, TRR was at 0%-3.6%, 0.5%-3.7%, and 1%-1.4% for ECD, PP and TP, respectively. The FPR was at 1.1%-1.8%, 1.1%-3.3%, 2.9%, respectively. TP was only analyzed in one prior meta-analysis, i.e., in the only prior network meta-analysis (22), limiting the robustness of comparative conclusions involving this technique. Hence, TRR and FPR were in the same range and ranking for the different surgery techniques in the present meta-analysis. While the presented meta-analysis broadly aligns with earlier findings for the comparison between ECD and PP, the inclusion of additional data on TP allows for exploratory comparisons across a wider range of surgical techniques. These results suggest that, in selected cases where ECD is feasible, TRR may be comparable to PP. Although a trend towards lower TRR after TP compared to ECD/PP was observed, this finding contrasts with a prior meta-analysis (21), and also with a previous network meta-analysis showing not a lower TRR after TP (22). Therefore, it should be interpreted with caution.

Importantly, these comparisons are subject to limitations, including substantial clinical heterogeneity and missing assessment of risk of bias in the included studies due to an already limited data situation. A fundamental assumption underlying the validity of indirect comparisons in a network meta-analysis is transitivity, which requires that patients included in the different pairwise comparisons forming the network are sufficiently similar with respect to clinically relevant effect modifiers – in particular, tumor size, tumor location, surgeon experience, and patient selection criteria. If transitivity is violated, the estimated indirect treatment effects may be biased regardless of statistical consistency. In the present analysis, transitivity is likely compromised: ECD is preferentially applied to small, mobile, superficial tumors by experienced surgeons, whereas PP covers a broader range of tumor sizes and locations, and TP is predominantly reserved for deep-lobe tumors, large lesions, or cases with diffuse glandular involvement. Consequently, the three techniques are applied to clinically distinct patient populations, and the assumption that patients across the respective pairwise comparisons are exchangeable – a prerequisite for valid indirect inference – cannot be upheld with the available data. This confounding by indication represents a key limitation of the network meta-analysis and must be considered when interpreting the indirect comparisons between ECD, PP, and TP. Statistical consistency testing, as performed in the present analysis, evaluates the coherence between direct and indirect estimates within the network but does not test transitivity and cannot substitute for it. The results of the network meta-analysis should therefore be regarded as exploratory. Additionally, the small number of studies on permanent facial paresis included in this analysis limits the reliability of Egger's test.

It is also important to consider that an unclear definition of PP could result in the intervention received being misclassified, thereby weakening the consistency and thus the indirect comparability of interventions. Therefore, the results should be interpreted with caution.

The results also show a low facial palsy rate after parotid surgery in general. This might be due to the general predominance of benign histopathological diagnoses in the included studies which is also consistent with the high rate of PP. This likely reflects the current clinical practice, in which less extensive surgical approaches are preferentially applied to benign and favorably located tumors, thereby minimizing the risk of facial injury. These findings highlight the close relationship between tumor pathology, surgical strategy and clinical outcome in parotid gland surgery, as also demonstrated in a large single-center series by Aliyeva. et al. (66).

## Conclusions

To be able to make meaningful statements about TRR and permanent FPR comparing different parotid surgery techniques for benign tumors, it was necessary to select only studies with sufficient follow-up and clear criteria for a permanent facial palsy. Due to the imprecision of the definition of several procedures resecting only parts of the parotid glands, it also made sense to consider them cumulatively as PP techniques. Under these conditions, 23 publications with 4,674 adult patients could be included into pairwise meta-analyses and into a network meta-analysis comparing the complete range of techniques from ECD over PP to TP. This combined pairwise and network meta-analysis suggest that, in selected cases where ECD is feasible, i.e., for mobile superficial small tumors, and in the hands of experienced surgeons, TRR may be comparable to PP. TRR seems to be lower after TP compared to ECD/PP but this trend needs to be interpreted carefully due to several limitations and missing data in long term follow-up. The present study shows tendencies for a low risk of permanent facial palsy but lacks data for facial palsy after TP. We need prospective randomized studies directly comparing at least ECD and PP with long-term follow-up to assess the benefits and risks with high evidence.

## Data Availability

The original contributions presented in the study are included in the article/Supplementary Material, further inquiries can be directed to the corresponding author.
