# Supplementary material for: Permanent facial palsy and recurrence rate after surgery for benign parotid tumors: pairwise and network meta-analysis of different parotid surgery techniques
Source: Front Surg. 2026 May 7;13:1836835. doi: 10.3389/fsurg.2026.1836835 (PMC13189896; doi:10.3389/fsurg.2026.1836835)

**Supplementary Tables and Figure**

**Permanent facial palsy and recurrence rate after surgery for benign parotid tumors: pairwise and network meta-analysis of different parotid surgery techniques**

Caroline Bernhard, Peter Schlattmann, Orlando Guntinas-Lichius

**Supplementary Table S1**

**Supplementary Table S2**

**Supplementary Table S3**

**Supplementary Table S4**

**Supplementary Table S5**

**Supplementary Table S6**

**Supplementary Table S7**

**Supplementary Figure S1**

**Supplementary Table S1**

| Supplementary Table S1. MeSH terms and combinations used to identify relevant studies. | | |
| --- | --- | --- |
| Search | **Terms** | **Hits** |
| 1 | parotid benign tumor*[Text Word] | 28 |
| 2 | parotid neoplasm*[Text Word] | 9711 |
| 3 | salivary gland neoplasm*[Text Word] | 10567 |
| 4 | parotid gland tumor*[Text Word] | 723 |
| 5 | ("parotid benign tumor*"[Text Word] OR "parotid neoplasm*"[Text Word] OR "salivary gland neoplasm*"[Text Word] OR "parotid gland tumor*"[Text Word]) | 19421 |
| 6 | Parotidectomy [Text Word] | 3321 |
| 7 | partial parotidectomy [Text Word] | 154 |
| 8 | lateral parotidectomy [Text Word] | 32 |
| 9 | superficial parotidectomy [Text Word] | 967 |
| 10 | total parotidectomy [Text Word] | 686 |
| 11 | (Parotid surgery[MeSH Terms]) OR (Salivary surgery[MeSH Terms]) OR (Parotidectomy[Text Word]) OR (partial parotidectomy[Text Word]) OR (lateral parotidectomy[Text Word]) OR (superficial parotidectomy[Text Word]) OR (total parotidectomy[Text Word])) OR (extracapsular dissection[Text Word]) | 13525 |
| 12 | (recurrence[Text Word]) | 604539 |
| 13 | (facial pals*[Text Word]) | 5700 |
| 14 | (facial paralysis[Text Word]) | 15565 |
| 15 | (facial paresis[Text Word]) | 767 |
| 16 | (FNP[Text Word]) | 662 |
| 17 | (recurrence[Text Word]) OR (facial pals*[Text Word]) OR (facial paralysis[Text Word]) OR (facial paresis[Text Word]) OR (FNP[Text Word]) | 622547 |
| 18 | ((((((Parotid benign tumor*[Text Word])) OR (Parotid Neoplasm*[Text Word])) OR (salivary gland neoplasm*[Text Word])) OR (parotid gland tumor*[Text Word])) AND ((((((((Parotid surgery[MeSH Terms]) OR (Salivary surgery[MeSH Terms])) OR (Parotidectomy[Text Word])) OR (partial parotidectomy[Text Word])) OR (lateral parotidectomy[Text Word])) OR (superficial parotidectomy[Text Word])) OR (total parotidectomy[Text Word])) OR (extracapsular dissection[Text Word]))) AND (((((recurrence[Text Word]) OR (facial pals*[Text Word])) OR (facial paralysis[Text Word])) OR (facial paresis[Text Word])) OR (FNP[Text Word])) | 1344 |

**Supplementary Table S2**

| **Supplementary Table S2**. Reasons for exclusion of studies during the full-text analysis. | | |
| --- | --- | --- |
| **Reason** | **Number of studies** |  |
| Study was not available in German or English | 12 |  |
| Study was not an randomized controlled trial (RCT), cohort or case-control study, but e.g. a review or a single case study | 5 |  |
| Similar parotid surgery techniques were compared | 18 |  |
| Incomplete or unclear information (e.g. follow-up duration not specified, unclear number of cases, no breakdown of the outcome by surgical method, facial nerve palsy not defined) | 28 |  |
| Selection criteria not met (e.g. population <10, no separation of malignant and benign tumors, surgical methods not explained or incorrectly defined, children included, facial nerve palsy examined without House-Brackmann (HB) score, follow-up too short, secondary operations included) | 39 |  |
| Sum of excluded studies | 102 |  |

**Supplementary Table S3**

| **Supplementary Table S3.** ROBINS-I assessment of risk of bias of the retrospective studies and one included prospective non-randomized study* | | | | |  |
| --- | --- | --- | --- | --- | --- |
| **Barca & Cristofaro (2020) – 15-year Retrospective Cohort Study** | | | | |  |
| **ROBINS-I Domain** | **Risk of Bias** | | **Justification** | |  |
| Bias due to confounding | **Moderate** | | Retrospective design without adjustment for potential confounders such as tumour size, surgeon experience, or patient comorbidities. Allocation between ECD and SP was randomised, partially mitigating this concern. | |  |
| Bias in selection of participants | **Low** | | All consecutive patients from a single centre with pleomorphic adenoma in the superficial lobe were included; explicit inclusion/exclusion criteria were applied, with at least 3-year follow-up required. | |  |
| Bias in classification of intervention | **Low** | | Surgical technique (ECD vs. SP) is clearly defined and consistently applied. Standardised diagnostic work-up (US, CT/MRI, FNAC) was performed preoperatively in all patients. | |  |
| Bias due to deviations from intended interventions | **Low** | | No evidence of systematic deviations from the intended intervention; both procedures follow established protocols. Intraoperative conversions are not discussed but were unlikely common. | |  |
| Bias due to missing data | **Low** | | Only patients with at least 3 years of follow-up were included, and a structured imaging schedule was maintained. Loss to follow-up is not explicitly quantified but appears minimal. | |  |
| Bias in measurement of outcomes | **Moderate** | | Outcome assessment (facial nerve injury, Frey syndrome, recurrence) was not blinded to treatment allocation. Reliance on clinical examination may underestimate Frey syndrome. | |  |
| Bias in selection of the reported result | **Moderate** | | Univariate analysis only; no adjustment for multiple comparisons. Selection of reported outcomes appears comprehensive, but the retrospective nature raises the possibility of outcome reporting bias. | |  |
| **Overall bias** | **Moderate** | | The retrospective design and lack of confounder adjustment yield a moderate overall risk of bias, partially offset by structured follow-up and clear outcome definitions. | |  |
| **Bonavolontà et al. (2019) – Long-term Follow-up of 297 Patients** | | | | |  |
| **ROBINS-I Domain** | **Risk of Bias** | | **Justification** | |  |
| Bias due to confounding | **Moderate** | | No multivariable adjustment for tumour size, which differed between groups (smaller lesions predominantly received ECD). This constitutes an important confounding variable affecting complication rates. | |  |
| Bias in selection of participants | **Moderate** | | Fourteen patients were excluded due to irregular follow-up or pre-existing facial palsy. Selection of the surgical technique was not randomised, raising the possibility of selection bias by tumour characteristics. | |  |
| Bias in classification of intervention | **Low** | | ECD and SP are clearly defined with detailed operative descriptions. Diagnostic criteria (US, CT/MRI, FNAC) were applied uniformly. Quer classification was used for case categorisation. | |  |
| Bias due to deviations from intended interventions | **Low** | | Both techniques were applied according to predefined criteria based on lesion size and location. No evidence of systematic crossover or protocol deviations is reported. | |  |
| Bias due to missing data | **Moderate** | | Fourteen patients were excluded due to loss to follow-up. The mechanism of loss is not fully characterised; differential drop-out between groups cannot be excluded. | |  |
| Bias in measurement of outcomes | **Moderate** | | Complications were documented at standardised follow-up visits, but outcome assessment was not blinded to treatment allocation. Frey syndrome assessment was based on clinical complaint, potentially underestimating true incidence. | |  |
| Bias in selection of the reported result | **Moderate** | | Chi-squared test was used for all comparisons; no adjustment for multiplicity. The paper comprehensively reports complications and recurrence, but subgroup analyses may have been selectively presented. | |  |
| **Overall bias** | **Moderate** | | Moderate overall risk due to non-randomised allocation, potential selection bias by tumour size, and unblinded outcome assessment, despite a large cohort and structured follow-up. | |  |
| **Cheng et al. (2020) – ECD in 144 Patients (Taiwan)** | | | | |  |
| **ROBINS-I Domain** | **Risk of Bias** | | **Justification** | |  |
| Bias due to confounding | **Moderate** | | Surgical technique was chosen based on surgeon preference and tumour location, introducing allocation bias. Tumour location at the parotid tail was a strong independent predictor of ECD, partially confounding outcome comparisons. | |  |
| Bias in selection of participants | **Moderate** | | Non-randomised, retrospective design at a single tertiary centre. Surgeon preference influenced technique selection; patients with tail tumours were more likely to receive ECD, potentially skewing complication rates. | |  |
| Bias in classification of intervention | **Low** | | ECD and SP are clearly defined. The minimum fascia-tumour distance was systematically measured by ultrasound. Pathological subtypes were confirmed histologically in all cases. | |  |
| Bias due to deviations from intended interventions | **Low** | | No evidence of systematic deviations from intended interventions; the surgical choice was made preoperatively and adhered to. Intraoperative conversion criteria are mentioned but not quantified. | |  |
| Bias due to missing data | **Low** | | Inclusion criteria are clearly stated; patients with malignant or non-neoplastic diagnoses were excluded. Follow-up was standardised (annual US); mean follow-up of 56 months is adequate. | |  |
| Bias in measurement of outcomes | **Moderate** | | Facial nerve function was graded by House-Brackmann scale, a validated tool. However, assessment was not blinded. Transient vs. permanent palsy was defined by a 12-month threshold, which is appropriate. | |  |
| Bias in selection of the reported result | **Moderate** | | Multivariate logistic regression was applied to assess technique choice, but primary outcome comparisons (complications, recurrence) rely on univariate analysis only. Selective reporting of subgroup results cannot be excluded. | |  |
| **Overall bias** | **Moderate** | | Moderate overall risk due to non-randomised allocation driven by tumour location, retrospective design, and unblinded outcome ascertainment. Systematic confounder adjustment was applied for technique selection but not for outcomes. | |  |
| **Committeri et al. (2023) – Retrospective Analysis 2012–2021 (554 Patients)** | | | | |  |
| **ROBINS-I Domain** | **Risk of Bias** | | **Justification** | |  |
| Bias due to confounding | **Serious** | | No adjustment for potential confounders including tumour size (ECD used for lesions ≤3 cm, SP for >3 cm or deep lobe). This systematic difference in case mix is a major confounding factor affecting all outcome comparisons. | |  |
| Bias in selection of participants | **Moderate** | | 169 of 723 patients were excluded (malignant histology, pre-existing facial palsy, incomplete follow-up). Non-random allocation based on tumour size and location introduces systematic differences between groups. | |  |
| Bias in classification of intervention | **Low** | | ECD and SP are defined in line with the European Salivary Gland Society classification; five levels of each technique are described. Tumour characterisation via US, MRI, and FNAC was performed uniformly. | |  |
| Bias due to deviations from intended interventions | **Low** | | Operations were performed by a mixed-experience surgical team (senior, mid-level, junior); 25% supervised by seniors. This variability in operator experience may have influenced outcomes but does not constitute a planned deviation. | |  |
| Bias due to missing data | **Moderate** | | 79 patients were excluded due to irregular follow-up. The differential between groups regarding loss to follow-up is not reported, limiting assessment of informative censoring. | |  |
| Bias in measurement of outcomes | **Moderate** | | Complications were classified by timing (early, mid-term, late) and documented at standardised follow-up intervals. Outcome assessment was not blinded to treatment allocation; Frey syndrome relied on patient-reported symptoms. | |  |
| Bias in selection of the reported result | **Moderate** | | Chi-squared test was used throughout. All major complications and recurrences are reported, but differential complication rates across surgeon experience levels are acknowledged without formal analysis. | |  |
| **Overall bias** | **Serious** | | Serious overall risk of bias driven by systematic case-mix differences between groups (tumour size), absence of confounder adjustment, and unblinded outcome assessment, making direct technique comparisons unreliable. | |  |
| **Cristofaro et al. (2014) – 10-year Retrospective Cohort Study** | | | | |  |
| **ROBINS-I Domain** | **Risk of Bias** | | **Justification** | |  |
| Bias due to confounding | **Moderate** | | Surgical technique was randomly assigned, which substantially reduces confounding. However, mean age and lesion size differed numerically between groups, and no multivariable adjustment was performed. | |  |
| Bias in selection of participants | **Low** | | Randomised allocation to ECD or SP from a single centre; consecutive patients with superficial-lobe pleomorphic adenoma were included with clearly stated criteria. Histological confirmation was obtained in all cases. | |  |
| Bias in classification of intervention | **Low** | | Both techniques are clearly described with standardised operative steps including facial nerve monitoring for SP. Diagnostic criteria (US, CT/MRI, FNAC) were uniformly applied. | |  |
| Bias due to deviations from intended interventions | **Low** | | Surgical technique was randomly assigned and adhered to according to predefined protocols. No deviations from intended interventions are reported. | |  |
| Bias due to missing data | **Low** | | A structured five-year follow-up protocol (monthly clinical exams, US, CT/MRI at set intervals) was applied. Patients were discharged after a mean of 3 days with no locoregional complications, indicating complete early follow-up. | |  |
| Bias in measurement of outcomes | **Moderate** | | Outcome assessment (facial nerve injury, recurrence, fistula) was performed by the clinical team without blinding. The use of a neurostimulator for nerve monitoring is a strength; Frey syndrome was not encountered, limiting its assessment. | |  |
| Bias in selection of the reported result | **Low** | | Both primary and secondary outcomes are fully reported with p-values. Univariate analysis only; however, the randomised allocation limits the impact of this on validity. | |  |
| **Overall bias** | **Moderate** | | Moderate overall risk due to lack of multivariable analysis and unblinded outcome assessment, substantially offset by randomised allocation and structured follow-up. | |  |
| **Dell'Aversana Orabona et al. (2013) – 232 Cases (Naples)** | | | | |  |
| **ROBINS-I Domain** | **Risk of Bias** | | **Justification** | |  |
| Bias due to confounding | **Moderate** | | Non-randomised allocation; ECD was used for smaller tumours (mean 1.89 cm) and SP for larger ones (mean 3.49 cm), introducing systematic case-mix differences. No adjustment for tumour size was performed. | |  |
| Bias in selection of participants | **Moderate** | | Retrospective review of consecutive patients; allocation by tumour size and location leads to systematic differences in baseline characteristics. No formal randomisation or matching was applied. | |  |
| Bias in classification of intervention | **Low** | | ECD and SP are clearly described with standardised incisions and operative steps. Histological subtypes (pleomorphic adenoma, Warthin tumour, myoepithelioma, lipoma) were confirmed for all cases. | |  |
| Bias due to deviations from intended interventions | **Low** | | Procedures followed predefined criteria (ECD for superficial lobe, SP for tumours >3 cm or deep lobe). No deviations from the planned intervention are described. | |  |
| Bias due to missing data | **Low** | | Follow-up ranged from 1 to 108 months (mean ~46–52 months). The retrospective design precludes certainty about completeness, but no loss to follow-up is explicitly reported. | |  |
| Bias in measurement of outcomes | **Moderate** | | Outcomes (facial nerve injury, Frey syndrome, recurrence, capsular rupture) were assessed clinically without blinding. Log-rank (Cox-Mantel) test was used, which is appropriate for time-to-event data. | |  |
| Bias in selection of the reported result | **Moderate** | | All major outcomes are reported. Statistical comparisons are limited to chi-squared and log-rank tests without adjustment for confounders such as tumour size, which differed significantly between groups. | |  |
| **Overall bias** | **Moderate** | | Moderate overall risk due to non-randomised allocation by tumour size, absence of confounder adjustment, and unblinded outcome ascertainment, despite comprehensive outcome reporting. | |  |
| **Guntinas-Lichius et al. (2004) – 13-year Standardised Parotidectomy Series** | | | | |  |
| **ROBINS-I Domain** | **Risk of Bias** | | **Justification** | |  |
| Bias due to confounding | **Low** | | All patients with pleomorphic adenoma received the same standardised lateral or total parotidectomy, eliminating between-technique confounding. Comparison is between lateral and total parotidectomy based on tumour location. | |  |
| Bias in selection of participants | **Moderate** | | Chart review of 295 of 424 patients; 129 records were unavailable, and only 171 (58%) answered the questionnaire. Questionnaire responders may differ systematically from non-responders. | |  |
| Bias in classification of intervention | **Low** | | Surgical technique was strictly standardised (microscope-assisted lateral or total parotidectomy) based on tumour location relative to the facial nerve fan. Classification is explicit and consistently applied. | |  |
| Bias due to deviations from intended interventions | **Low** | | All operations followed the same standardised protocol with no planned deviations. The use of an operating microscope and visual facial monitoring (transparent plastic film) was consistent. | |  |
| Bias due to missing data | **Serious** | | Only 58% of patients answered the questionnaire; 67 patients who reported complications were re-examined but only 62 attended. Differential non-response related to outcomes introduces serious risk of informative censoring. | |  |
| Bias in measurement of outcomes | **Moderate** | | Facial nerve function was objectively assessed by needle electromyography; Frey syndrome by Minor's iodine-starch test in re-examined patients. However, questionnaire-based self-reporting for the larger cohort is subject to recall bias. | |  |
| Bias in selection of the reported result | **Moderate** | | Disease-free survival calculated by Kaplan-Meier method. Primary outcomes are fully reported. Discrepancy between questionnaire-reported and objectively confirmed complication rates (e.g., Frey syndrome 54% vs. 21%) is acknowledged. | |  |
| **Overall bias** | **Moderate** | | Moderate overall risk due to substantial questionnaire non-response and differential re-examination, partially offset by objective outcome verification in re-examined patients and highly standardised surgical technique. | |  |
| **Iro et al. (2013) – Follow-up of ECD for Pleomorphic Adenoma (Erlangen)** | | | | |  |
| **ROBINS-I Domain** | **Risk of Bias** | | **Justification** | |  |
| Bias due to confounding | **Low** | | This study reports exclusively on ECD patients; no between-technique comparison is made for the primary outcome (recurrence). Confounding is therefore not applicable for the primary analysis. | |  |
| Bias in selection of participants | **Moderate** | | 76 of 219 pleomorphic adenoma cases received ECD; case selection was based on preoperative clinical and sonographic criteria (single, mobile, superficial tumour). Cases selected for ECD may represent a favourable subgroup. | |  |
| Bias in classification of intervention | **Low** | | ECD is precisely defined (removal without exposure of the facial nerve main trunk) with a four-stage classification system clearly distinguishing it from parotidectomy variants. All cases were histologically confirmed. | |  |
| Bias due to deviations from intended interventions | **Low** | | Intraoperative decision to convert from ECD to parotidectomy was explicitly permitted and described. Neuromonitoring was mandatory; operative technique was standardised across all cases. | |  |
| Bias due to missing data | **Moderate** | | 18 of 76 patients (23.7%) could not attend in person and were assessed remotely by ENT colleagues. While written reports were obtained, remote assessment may differ in quality from direct clinical and sonographic examination. | |  |
| Bias in measurement of outcomes | **Low** | | Follow-up included annual clinical examination and sonography; mean follow-up was 7.38 years (range 5.05–10.52). Recurrence (primary outcome) was assessed by both clinical and imaging criteria, which are objective. | |  |
| Bias in selection of the reported result | **Low** | | The primary outcome (recurrence rate) is clearly pre-specified and fully reported. All cases are accounted for. Comparison with parotidectomy groups is presented descriptively with appropriate caveats. | |  |
| **Overall bias** | **Low** | | Low overall risk for the primary outcome (recurrence after ECD) given objective outcome assessment, long follow-up, and complete case ascertainment. Moderate risk for secondary complication comparisons due to non-randomised technique selection. | |  |
| **Kadletz et al. (2020) – Cross-sectional Study on Frey's Syndrome** | | | | |  |
| **ROBINS-I Domain** | **Risk of Bias** | | **Justification** | |  |
| Bias due to confounding | **Serious** | | Patients treated with ECD were significantly younger (mean 37.6 vs. 49.0 years) and had longer follow-up; these factors independently influence Frey syndrome risk. No adjustment for age or follow-up duration was performed. | |  |
| Bias in selection of participants | **Serious** | | Only 127 of 636 patients (20%) were contactable and agreed to participate. The ratio of ECD to SP was 1:2, reflecting historical practice patterns. This severe selection may introduce substantial bias. | |  |
| Bias in classification of intervention | **Low** | | ECD and SP are defined according to the European Salivary Gland Society criteria. All histological diagnoses (pleomorphic adenoma, Warthin tumour) were confirmed pathologically. | |  |
| Bias due to deviations from intended interventions | **Low** | | No evidence of deviation from intended surgical procedures; categorisation was based on original operative reports, providing an objective classification source. | |  |
| Bias due to missing data | **Serious** | | Only 20% of the original cohort was assessable. Non-participation may be systematically related to outcomes (e.g., patients with complications more or less likely to respond), introducing serious informative dropout. | |  |
| Bias in measurement of outcomes | **Moderate** | | Outcomes were assessed by structured telephone/mail questionnaire without objective examination (no Minor's iodine-starch test). Patient recall over a mean follow-up of 21.3 years is subject to recall bias; Frey syndrome may be underreported. | |  |
| Bias in selection of the reported result | **Moderate** | | Fisher's exact test was used for all comparisons. All pre-specified outcomes are reported. However, the absence of multivariable analysis given substantial baseline differences renders reported associations potentially misleading. | |  |
| **Overall bias** | **Serious** | | Serious overall risk of bias due to extreme selection bias (20% response rate), major baseline confounding by age and follow-up duration, and subjective outcome assessment without examination over very long recall periods. | |  |
| **Laskaris et al. (2022) – PSP vs. ECD for Benign Parotid Tumours (Greece)** | | | | |  |
| **ROBINS-I Domain** | **Risk of Bias** | | **Justification** | |  |
| Bias due to confounding | **Moderate** | | Non-randomised allocation; tumour size and location were not used as selection criteria, which the authors note as a limitation. Differences in sex distribution (79.7% female in ECD vs. 44% in SP) may confound outcomes. | |  |
| Bias in selection of participants | **Moderate** | | Retrospective, non-randomised design at a single ENT centre. Patients with capsular rupture intraoperatively were excluded, which may selectively remove higher-risk ECD cases and underestimate its complication rate. | |  |
| Bias in classification of intervention | **Low** | | PSP and ECD are clearly defined with standardised operative steps. Nerve stimulation was used in both groups. All tumours were confirmed benign by histology postoperatively. | |  |
| Bias due to deviations from intended interventions | **Low** | | Both procedures were performed by experienced surgeons following defined protocols. No deviations from intended interventions are reported; capsule rupture cases were excluded from analysis. | |  |
| Bias due to missing data | **Moderate** | | Follow-up extended to 2 years with structured visits at 1 week, 6 months, 1 year, and 2 years. The 2-year follow-up is insufficient to reliably detect pleomorphic adenoma recurrence, which may appear years later. | |  |
| Bias in measurement of outcomes | **Moderate** | | Facial nerve function was assessed by clinical signs and electromyography; Frey syndrome and fistula were clinically documented. Outcome assessment was not blinded to treatment allocation. | |  |
| Bias in selection of the reported result | **Moderate** | | Pearson's chi-square test was used without confounder adjustment. All pre-specified outcomes are reported. The short follow-up severely limits the validity of the recurrence comparison. | |  |
| **Overall bias** | **Moderate** | | Moderate overall risk due to non-randomised allocation, sex imbalance between groups, short follow-up inadequate for recurrence assessment, and unblinded outcome ascertainment. | |  |
| **Laskawi et al. (1996) – Follow-up Study of Three Methods (475 Patients)** | | | | |  |
| **ROBINS-I Domain** | **Risk of Bias** | | **Justification** | |  |
| Bias due to confounding | **Moderate** | | Non-randomised allocation of surgical technique based on tumour location (superficial vs. deep lobe) rather than patient or surgeon preference. Deep lobe tumours received total parotidectomy, introducing case-mix differences. | |  |
| Bias in selection of participants | **Moderate** | | Retrospective chart review; follow-up data were available for only 223 of 475 patients (47%) via questionnaire to referring doctors. Patients with complete follow-up may differ from those lost to follow-up. | |  |
| Bias in classification of intervention | **Low** | | Three techniques (superficial parotidectomy, total parotidectomy, enucleation) are clearly defined. Persistent facial nerve paresis was defined as lasting >6 months, which is an explicit and appropriate threshold. | |  |
| Bias due to deviations from intended interventions | **Low** | | Surgical technique was allocated based on pre-defined anatomical criteria (tumour location). Enucleation was reserved for exceptional cases. No evidence of systematic deviations from intended procedures. | |  |
| Bias due to missing data | **Serious** | | Follow-up data were obtained for only 47% of the original cohort via questionnaire to referring doctors, who may have incomplete information. This large loss to follow-up introduces serious risk of informative censoring. | |  |
| Bias in measurement of outcomes | **Moderate** | | Outcomes were assessed by questionnaire to referring doctors, introducing potential for recall and reporting bias. Facial nerve function was classified by a >6-month threshold, which is appropriate but not verified objectively. | |  |
| Bias in selection of the reported result | **Moderate** | | All three techniques and their outcomes are reported. Only one recurrence occurred in each of the SP and enucleation groups, limiting statistical inference. No adjustment for confounders was performed. | |  |
| **Overall bias** | **Serious** | | Serious overall risk due to loss of more than half the cohort to follow-up, questionnaire-based outcome assessment without objective verification, and small numbers of events limiting meaningful statistical comparison. | |  |
| **Lee et al. (2017) – ECD for Warthin Tumour in the Parotid Tail** | | | | |  |
| **ROBINS-I Domain** | **Risk of Bias** | | **Justification** | |  |
| Bias due to confounding | **Moderate** | | Non-randomised allocation; surgical technique was determined by preoperative FNAC and CT findings. Although demographic characteristics were similar, the basis for technique selection may introduce residual confounding. | |  |
| Bias in selection of participants | **Moderate** | | Retrospective review of 72 patients from 562 who underwent parotid surgery; selection for inclusion was based on Warthin tumour location in the tail. The representativeness of this subset is unclear. | |  |
| Bias in classification of intervention | **Low** | | ECD and SP are explicitly defined (with and without facial nerve trunk/branch identification, respectively). Intraoperative facial nerve monitoring was routinely used. All diagnoses were histopathologically confirmed. | |  |
| Bias due to deviations from intended interventions | **Low** | | One patient in the ECD group was a revision case (re-operated after recurrence following SP) and facial nerve palsy during this re-operation was appropriately excluded from the primary ECD complication count. | |  |
| Bias due to missing data | **Moderate** | | Follow-up ranged from 3 to 116 months (mean ECD: 30.9 months, SP: 73.4 months). The shorter follow-up in the ECD group limits comparability of recurrence rates; the study authors acknowledge this limitation. | |  |
| Bias in measurement of outcomes | **Low** | | Facial nerve function was assessed by House-Brackmann grade by clinicians; all complications including haematoma and salivary fistula were documented from medical records. Recurrence was confirmed clinically and radiologically. | |  |
| Bias in selection of the reported result | **Moderate** | | Fisher's exact test was used for categorical comparisons. Operation time was compared with independent t-test. All pre-specified outcomes are reported; however, unequal follow-up between groups biases the recurrence comparison. | |  |
| **Overall bias** | **Moderate** | | Moderate overall risk due to non-randomised allocation, significantly different follow-up durations between groups, and retrospective design, partially offset by histological confirmation and objective complication assessment. | |  |
| **Mantsopoulos et al. (2018) – Multifocal Cystadenolymphomas (Erlangen)** | | | | |  |
| **ROBINS-I Domain** | **Risk of Bias** | | **Justification** | |  |
| Bias due to confounding | **Moderate** | | Multifocal extracapsular dissection (MED) was preferentially selected for conglomerate lesions in the caudal pole, while complete parotidectomy (CP) was chosen for more diffuse or demanding cases. This case-mix difference is a potential confounder acknowledged by the authors. | |  |
| Bias in selection of participants | **Moderate** | | Retrospective analysis at a single tertiary referral centre. The number of MED cases increased substantially over the study period (from virtually zero pre-2006 to >10/year post-2011), introducing a temporal trend that may confound outcomes. | |  |
| Bias in classification of intervention | **Low** | | MED and CP are precisely defined; multifocality is defined histologically (>1 focus on definitive histology) or by separate intraoperative resection of multiple lesions. Definitions are clear and consistently applied. | |  |
| Bias due to deviations from intended interventions | **Low** | | Surgical technique was selected according to predefined criteria at each institution. No deviations from intended procedures are described; standardised preoperative imaging (US, occasionally CT/MRI) was performed in all cases. | |  |
| Bias due to missing data | **Moderate** | | Mean follow-up was 60 months; the limited follow-up in earlier years (fewer MED cases 2000–2009) may underestimate metachronous tumour rates in the MED group. Authors acknowledge this as a limitation. | |  |
| Bias in measurement of outcomes | **Moderate** | | Permanent facial palsy was assessed clinically (House-Brackmann) and by electromyography. Frey syndrome was assessed by clinical report. Outcome assessment was not blinded to treatment allocation. | |  |
| Bias in selection of the reported result | **Moderate** | | Chi-square test with 95% confidence intervals was used. All major outcomes (metachronous tumour rate, facial palsy, Frey syndrome, fistula, haemorrhage) are reported. The temporal trend in technique adoption limits interpretability. | |  |
| **Overall bias** | **Moderate** | | Moderate overall risk due to systematic case selection bias (easier cases receiving MED), temporal confounding, and limited follow-up for earlier MED cases, partially offset by precise outcome definitions and comprehensive reporting. | |  |
| **Ozturk et al. (2019) – Comparative Outcomes of ECD and SP** | | | | |  |
| **ROBINS-I Domain** | **Risk of Bias** | | **Justification** | |  |
| Bias due to confounding | **Moderate** | | Non-randomised design with tumour characteristics (size <4 cm, single, superficial lobe) used as inclusion criteria rather than as allocation criteria, improving comparability. However, no multivariable adjustment was performed. | |  |
| Bias in selection of participants | **Moderate** | | 136 of 318 patients met inclusion criteria; the basis for exclusion of the remaining 182 patients is not fully described. ECD was performed by surgeons with >15 years of experience, potentially introducing operator selection bias. | |  |
| Bias in classification of intervention | **Low** | | ECD and SP are clearly described. Facial nerve neuromonitoring with two channels was used in all cases. Inclusion required pathologically proven benign FNAC, absence of deep lobe invasion, and single tumour <4 cm. | |  |
| Bias due to deviations from intended interventions | **Low** | | All operators had >15 years of experience in parotid surgery. Standardised incision and dissection protocols are described for both techniques. No deviations from intended interventions are reported. | |  |
| Bias due to missing data | **Moderate** | | Mean follow-up was 42.53 months (range 25–85 months). This duration is insufficient to reliably detect late pleomorphic adenoma recurrences. The mechanism and pattern of loss to follow-up are not described. | |  |
| Bias in measurement of outcomes | **Moderate** | | Facial nerve function was graded by House-Brackmann scale; Frey syndrome and complications were documented from clinic charts and follow-up forms. Outcome assessment was retrospective and not blinded. | |  |
| Bias in selection of the reported result | **Moderate** | | Fisher's exact test and independent t-test were used; no adjustment for confounders. All pre-specified outcomes are reported. The absence of any ECD complications may partly reflect relatively short follow-up and highly selected patients. | |  |
| **Overall bias** | **Moderate** | | Moderate overall risk due to non-randomised design, partial exclusion reporting, short follow-up for recurrence assessment, and unblinded outcome ascertainment, partially offset by strict inclusion criteria and uniform operator experience. | |  |
| **Prichard et al. (1992) – SP vs. Extracapsular Lumpectomy (Leicester)** | | | | |  |
| **ROBINS-I Domain** | **Risk of Bias** | | **Justification** | |  |
| Bias due to confounding | **Moderate** | | Non-randomised allocation; ECD was applied predominantly to tail lesions (90%), while SP was used for body and deep lobe tumours. Tumour location is a potential confounder for both complications and recurrence. | |  |
| Bias in selection of participants | **Moderate** | | Small series of 46 patients; technique was determined by tumour location and surgeon judgement. An independent surgeon assessed morbidity retrospectively, which strengthens outcome assessment but does not eliminate selection bias. | |  |
| Bias in classification of intervention | **Low** | | Extracapsular lumpectomy (ECD) and superficial parotidectomy are clearly distinguished. The paper explicitly contrasts ECD with enucleation, noting that a cuff of normal tissue is removed in ECD. All tumours were histologically characterised. | |  |
| Bias due to deviations from intended interventions | **Low** | | Operative procedures followed standard protocols; a nerve stimulator was available for identification of nerve branches as needed. No systematic deviations from intended interventions are described. | |  |
| Bias due to missing data | **Moderate** | | Mean follow-up was 54 months (median 46 months); minimum 2 years. This duration, while adequate for short-term recurrence detection, may miss late recurrences of pleomorphic adenoma, which can occur >10 years postoperatively. | |  |
| Bias in measurement of outcomes | **Low** | | Morbidity was assessed by an independent surgeon not involved in the primary surgery, reducing assessor bias. Frey syndrome was clinically diagnosed; facial palsy was classified as temporary or permanent. | |  |
| Bias in selection of the reported result | **Moderate** | | All major complications are reported for both groups. Statistical analysis is limited to description given the small sample. The small size severely limits statistical power and increases the risk of chance findings. | |  |
| **Overall bias** | **Moderate** | | Moderate overall risk due to non-randomised allocation by tumour location, small sample size limiting statistical inference, and follow-up potentially insufficient for late recurrence detection, partially offset by independent outcome assessment. | |  |
| **Riad et al. (2011) – Variables Related to Recurrence of Pleomorphic Adenomas** | | | | |  |
| **ROBINS-I Domain** | **Risk of Bias** | | **Justification** | |  |
| Bias due to confounding | **Moderate** | | This study prospectively examines variables predicting recurrence using logistic regression, appropriately adjusting for multiple potential confounders. However, the type of parotidectomy was determined by tumour characteristics, limiting causal inference. | |  |
| Bias in selection of participants | **Moderate** | | Data from three tertiary referral centres were analysed prospectively; 18 revision parotidectomies were included alongside 164 primary cases, introducing heterogeneity. Parapharyngeal tumours (n=22) were included, which differ substantially from superficial-lobe tumours. | |  |
| Bias in classification of intervention | **Low** | | Four surgical procedures are precisely defined (hemisuperficial, superficial, total, and revision parotidectomy) with explicit criteria for each. Tumour puncture was assessed histologically, which is an objective and validated measure. | |  |
| Bias due to deviations from intended interventions | **Low** | | Surgical technique was allocated based on pre-defined criteria (tumour size, location, recurrence status). Logistic regression was used to identify independent predictors, appropriately accounting for interrelated variables. | |  |
| Bias due to missing data | **Low** | | Follow-up ranged from 34 to 93 months (mean 56.4 months) and appears complete; the prospective design facilitated structured data collection. All 182 patients are included in the analysis. | |  |
| Bias in measurement of outcomes | **Low** | | Tumour puncture was assessed histologically (objective); tumour spillage was recorded intraoperatively. Safety margins were measured histologically. These objective measures substantially reduce measurement bias. | |  |
| Bias in selection of the reported result | **Low** | | Logistic regression was used to identify independent predictors of recurrence with explicit reporting of odds ratios, p-values, and confidence intervals. All analysed variables and outcomes are fully reported. | |  |
| **Overall bias** | **Low** | | Low overall risk for the primary aim (identifying predictors of recurrence) given prospective design, objective outcome measures, and appropriate multivariable analysis. Moderate risk for technique comparisons given non-randomised allocation. | |  |
| **Schapher et al. (2019) – ESGS Classification and Long-term Outcomes (Erlangen)** | | | | |  |
| **ROBINS-I Domain** | **Risk of Bias** | | **Justification** | |  |
| Bias due to confounding | **Moderate** | | Non-randomised; ED was preferentially used for smaller, more superficial tumours (ESGS categories I–II), while more extensive surgery was applied for larger lesions. Tumour size and ESGS category are confounders for all outcome comparisons. | |  |
| Bias in selection of participants | **Moderate** | | Complete follow-up was available for only 138 of 205 patients (67.3%); 67 patients were excluded primarily due to follow-up performed elsewhere or loss of contact. Non-completion may be related to outcomes. | |  |
| Bias in classification of intervention | **Low** | | Surgical techniques are classified according to the ESGS system; ED is defined with the established 2–4 mm margin criterion without facial nerve trunk exposure. FNAC and imaging were performed preoperatively in all cases. | |  |
| Bias due to deviations from intended interventions | **Low** | | Intraoperative decision to convert from ED to more extensive surgery was explicitly permitted when the facial nerve was encountered; this is appropriately described. Neuromonitoring was performed in all cases. | |  |
| Bias due to missing data | **Moderate** | | Follow-up required a minimum of 5 years (mean 7.7 years, maximum 11.7 years). All 138 included patients were seen at least once at ≥5 years. The 32.7% exclusion due to incomplete follow-up introduces potential informative censoring. | |  |
| Bias in measurement of outcomes | **Low** | | Recurrences were detected by ultrasound (primary imaging) in all cases; MRI was used in equivocal cases. Facial nerve paresis was assessed by House-Brackmann grading; Frey syndrome by standardised questioning. Objective methods are used. | |  |
| Bias in selection of the reported result | **Low** | | Chi-square test was used for categorical comparisons; all pre-specified ESGS categories, surgical techniques, and outcomes (FNP, Frey syndrome, recurrence) are fully reported. The ESGS classification framework improves comparability. | |  |
| **Overall bias** | **Moderate** | | Moderate overall risk due to non-randomised technique allocation by tumour size/category, 32.7% exclusion for incomplete follow-up, and potential residual confounding, partially offset by objective outcome assessment and long follow-up. | |  |
| **Uyar et al. (2011) – ECD vs. SP in Pleomorphic Adenomas (Long-term Follow-up)** | | | | |  |
| **ROBINS-I Domain** | **Risk of Bias** | | **Justification** | |  |
| Bias due to confounding | **Moderate** | | Non-randomised allocation; the same surgeon performed all operations with technique selection based on clinical and radiological evaluation. No formal adjustment for case-mix differences between groups was performed. | |  |
| Bias in selection of participants | **Moderate** | | Small series of 41 patients from a single centre; technique allocation was by surgeon judgement without randomisation. Both groups had similar demographic characteristics, but formal comparability assessment is limited by the small sample. | |  |
| Bias in classification of intervention | **Low** | | ECD and SP are explicitly defined with standardised incision techniques; an operating microscope and loupe were used for ECD. Frozen section of capsule was performed intraoperatively in all cases to verify margins. | |  |
| Bias due to deviations from intended interventions | **Low** | | All operations were performed by the same surgeon following consistent protocols. Facial nerve monitoring was used; if the tumour was large, the adjacent nerve branch was identified distally to proximally during ECD. | |  |
| Bias due to missing data | **Low** | | Mean follow-up was 194 months (range 117–264 months), one of the longest in the literature. All patients were followed clinically and radiologically. No loss to follow-up is reported, and both clinical and imaging monitoring were systematic. | |  |
| Bias in measurement of outcomes | **Moderate** | | Complications were assessed clinically; cosmetic deformity was evaluated by clinical inspection. Outcome assessment was not blinded. The assessment of Frey syndrome relied on clinical examination and patient complaint. | |  |
| Bias in selection of the reported result | **Moderate** | | One-way ANOVA was used for comparisons; chi-square and related tests were applied for categorical data. All outcomes including cosmetic deformity (often under-reported in the literature) are reported. No adjustments for multiplicity. | |  |
| **Overall bias** | **Moderate** | | Moderate overall risk due to non-randomised allocation, small sample size, and unblinded outcome assessment, substantially offset by exceptionally long follow-up, consistent single-surgeon technique, and intraoperative margin verification. | |  |
| **Vanroose et al. (2023) – Single-centre retrospective study, 161 patients (ECD vs. SP)** | | | | | |
| **ROBINS-I Domain** | | **Risk of Bias** | | **Justification** | |
| **Bias due to confounding** | | **Serious** | | Selection of ECD vs. SP was based on tumour size, location, and surgeon preference rather than randomisation. SP patients had significantly larger lesions (76 vs. 52 cm³; p=0.007) and longer follow-up (4.98 vs. 4.19 years; p=0.036). These imbalances constitute important confounders that are acknowledged but not adjusted for in the analysis. | |
| **Bias in selection of participants** | | **Moderate** | | Inclusion criteria were clearly defined (FNAC, postoperative pathology, ≥1 year follow-up). Total parotidectomy, malignant, and deep-lobe cases were appropriately excluded. However, a single-centre retrospective design introduces the risk of referral or case-mix bias; patients treated at a tertiary centre may not represent the broader population. | |
| **Bias in classification of intervention** | | **Low** | | The two surgical techniques (ECD and SP) are well described and mutually exclusive. Operative reports and histopathology reports were used to allocate cases. The distinction between techniques is unambiguous and consistently applied throughout the study period. | |
| **Bias due to deviations from intended interventions** | | **Moderate** | | No formal protocol for intraoperative switching between procedures is described, although the authors acknowledge that surgeons should be able to deviate from planned ECD when intraoperative findings require it. Any unplanned conversions are not systematically reported, which may introduce imprecision in group assignment. | |
| **Bias due to missing data** | | **Low** | | Inclusion criteria required complete preoperative FNAC and postoperative pathology data for all patients. Follow-up duration and clinical outcomes are reported for all 161 included cases. No mention of data loss or significant missing values is made; retrospective file completeness appears acceptable. | |
| **Bias in measurement of outcomes** | | **Moderate** | | Facial nerve weakness and Frey syndrome were assessed clinically without validated grading scales (e.g. House–Brackmann, starch-iodine test) as acknowledged by the authors. Outcomes were evaluated during routine follow-up consultations, introducing potential for differential detection bias between the more intensively followed SP group. | |
| **Bias in selection of the reported result** | | **Moderate** | | The study reports a comprehensive set of pre-specified outcomes. Cost-effectiveness was assessed via surrogate endpoints (operative time, anaesthesia time, length of stay) rather than actual monetary costs, which the authors acknowledge as a limitation. The outcome of recurrence is likely underreported given the short follow-up relative to biologically relevant recurrence windows (>10 years). | |
| **Overall bias** | | **Serious** | | The study is well conducted for a retrospective single-centre cohort, but the non-randomised design with significant prognostic imbalance between groups (tumour size, follow-up duration, sex distribution) and the absence of validated outcome measurement scales constitute serious concerns that limit causal inference. Results should be interpreted as exploratory. | |
| **Visconti et al. (2021) – Single-centre retrospective study, 540 patients (PSP vs. En vs. ECD)** | | | | | |
| **ROBINS-I Domain** | | **Risk of Bias** | | **Justification** | |
| **Bias due to confounding** | | **Serious** | | The allocation of patients to PSP, enucleation (En), or ECD was not randomised. ECD and En were reserved for selected cases with superficial, mobile lesions ≤1 cm, creating substantial prognostic imbalance. Patients undergoing PSP therefore had systematically different (larger, more complex) tumours, making direct outcome comparisons between techniques inherently confounded. | |
| **Bias in selection of participants** | | **Moderate** | | The study enrolled a large consecutive series (651 initially screened, 540 included) from a single institution over 16 years. Exclusion of patients lost to follow-up (n=27) and those with incomplete data introduces a potential attrition-related selection bias. The very long inclusion period also raises concerns about changes in case-mix and referral patterns over time. | |
| **Bias in classification of intervention** | | **Low** | | The three surgical approaches (PSP level I, PSP level II, En, ECD) are clearly defined according to the European Salivary Gland Society classification. Surgical technique is documented prospectively at the time of the procedure, and allocation to groups appears consistent and verifiable from operative records. | |
| **Bias due to deviations from intended interventions** | | **Low** | | All procedures were performed by the same surgical team using standardised equipment (Zeiss loupes, neuromapping, Shah's thermal scalpel) and a common surgical protocol. No deviations from intended technique or unplanned conversions are reported. The standardisation of the surgical approach across the study period is a notable methodological strength. | |
| **Bias due to missing data** | | **Moderate** | | A total of 27 patients (approximately 5%) were lost to follow-up and excluded from the analysis. The reasons for loss to follow-up are not described, and it is unclear whether attrition was differential across technique groups. Recurrence data are based on the latest available follow-up, which may underestimate true rates if losses were non-random. | |
| **Bias in measurement of outcomes** | | **Moderate** | | Facial nerve function and Frey syndrome were evaluated clinically by the operating team without blinding, using non-standardised assessments. The absence of the House–Brackmann scale and starch-iodine testing is acknowledged implicitly. The very long follow-up (mean 10.8 years) strengthens recurrence assessment, but subjective outcome ascertainment introduces measurement imprecision. | |
| **Bias in selection of the reported result** | | **Low** | | Outcomes are comprehensively reported across all three technique groups and across two time periods (2004–2012 vs. 2013–2020). Both favourable and unfavourable results (e.g. higher ECD recurrence rate) are transparently presented. The temporal sub-analysis of Frey syndrome by period appears pre-planned and adds valuable insight into the learning-curve effect. | |
| **Overall bias** | | **Serious** | | The fundamental methodological limitation is the intentional restriction of ECD and En to small, superficial, easily accessible lesions while PSP was applied to more complex tumours. This selection bias makes cross-technique comparisons unreliable. Despite the large sample size and long follow-up, the absence of randomisation and the unbalanced case-mix render the overall risk of bias serious. | |
| **Zheng et al. (2018) – Multi-centre retrospective study, 281 patients (SP vs. PSP vs. US-MECD)** | | | | | |
| **ROBINS-I Domain** | | **Risk of Bias** | | **Justification** | |
| **Bias due to confounding** | | **Moderate** | | Patient allocation across the three groups (SP, PSP, US-MECD) was not randomised; however, the groups show a high degree of comparability at baseline with no significant differences in age, sex, tumour size, histology, location, or follow-up duration (all p>0.05). Residual confounding by unmeasured factors (e.g. surgeon experience, case selection criteria per centre) cannot be excluded in this multi-centre design. | |
| **Bias in selection of participants** | | **Moderate** | | Patients were recruited from three hospitals with explicit inclusion (histologically confirmed benign superficial tumour ≤4 cm) and exclusion criteria (bilateral disease, prior surgery, systemic comorbidities). The multi-centre retrospective design limits verification of consistent case ascertainment across sites. The absence of a flow diagram makes it unclear how many patients were screened and excluded at each centre. | |
| **Bias in classification of intervention** | | **Low** | | All three surgical techniques are described in sufficient procedural detail to ensure unambiguous classification. The use of the ultrasonic scalpel (US-MECD) was restricted to a clearly defined patient group and documented separately. Surgical technique was determined prior to and independent of outcomes; misclassification is unlikely. | |
| **Bias due to deviations from intended interventions** | | **Low** | | The study states that all surgeons were highly experienced and trained within the same system, and that the same standard of surgery was maintained across the three participating hospitals. All three techniques were performed at each site. There is no report of protocol deviations or unintended crossovers between treatment groups. | |
| **Bias due to missing data** | | **Low** | | Complete outcome data appear to be available for all 281 included patients. Postoperative complications were assessed at pre-specified time points (in-hospital, 1, 3, 6, and 12 months). Recurrence was assessed in a defined subset (149 patients treated in 2012–2013) with comparable follow-up across groups. No significant missing data are reported. | |
| **Bias in measurement of outcomes** | | **Moderate** | | Facial nerve function was assessed using the validated House–Brackmann scale, which is a methodological strength. However, Frey syndrome was evaluated purely by subjective patient report without the Minor starch-iodine test, and peri-auricular sensation by a wisp of cotton/pinprick, both acknowledged as underestimating true complication rates. Outcome assessors were not reported to be blinded to treatment allocation. | |
| **Bias in selection of the reported result** | | **Low** | | A broad and pre-specified set of intraoperative and postoperative outcomes is reported for all three groups, including both favourable (recurrence, nerve preservation) and unfavourable outcomes (capsule rupture in US-MECD). Logistic regression analysis was pre-specified. Recurrence was analysed in a separate, clearly defined subset with adequate follow-up. No selective outcome reporting is apparent. | |
| **Overall bias** | | **Moderate** | | This multi-centre retrospective study is methodologically robust for an observational design, with well-matched groups, standardised surgical protocols, validated instruments for key outcomes, and comprehensive reporting. The main sources of residual bias are the non-randomised allocation, subjective assessment of Frey syndrome and sensation, and a follow-up period insufficient to capture late pleomorphic adenoma recurrence. Overall bias is rated moderate. | |

*Assessment using this Excel tool: Huang, XT, Automatic tool for Risk of Bias In Non-randomised Studies of Interventions (ROBINS-I), 2025. National Drug and Alcohol Research Centre (NDARC), University of New South Wales, Australia. Available from: https://www.unsw.edu.au/research/ndarc/resources/risk_of_bias_tool

Color coding: Low Moderate Serious

ECD = extracapsular dissection; SP = superficial parotidectomy; PSP = partial superficial parotidectomy; En = enucleation; US-MECD = ultrasonic scalpel-assisted minimal extracapsular dissection.

**Supplementary Table S4**

| **Supplementary Table S4.** Risk of Bias Assessment Using the Revised Cochrane Risk-of-Bias Tool for Randomized Trials (RoB 2) for two included prospective randomized trials. | | | |
| --- | --- | --- | --- |
| **Domain** | **Signalling Questions / Judgement Criteria** | **Rafi et al. [**[**60**](#_ENREF_60)**]**  **(PP vs ECD, n=46)** | **Abdwahed et al. [**[**46**](#_ENREF_46)**]**  **(PP vs ECD, n=50)** |
| **Domain 1: Bias arising from the randomization process** | | | |
| **1.1 Allocation sequence generation** | Was the allocation sequence random? | Unclear – method not explicitly described ("allocated" without specifying method) | Yes – Sealed-envelope (closed-envelope) random allocation described |
| **1.2 Allocation concealment** | Was the allocation sequence concealed until participants were enrolled and assigned to interventions? | No information provided on allocation concealment | Partial – sealed-envelope method implies some concealment, but intra-operative conversion of 3 ECD→PP patients raise concern |
| **1.3 Baseline differences** | Did baseline differences between groups suggest a problem with the randomization process? | Baseline characteristics (age, sex, tumor type) appear comparable; no p-values reported for baseline | No significant baseline differences; p-values for age, sex, pathology all >0.05 (Table 1) |
|  | **Domain 1 Verdict →** | **Some concerns** | **Some concerns** |
| **Domain 2: Bias due to deviations from intended interventions** | | | |
| **2.1 Blinding of participants and personnel** | Were participants and personnel blinded to intervention assignment? | No blinding reported; surgical procedures inherently unblinded (open-label surgical RCT) | No blinding reported; inherent to surgical intervention design |
| **2.2 Protocol deviations** | Were there deviations from the intended intervention that arose because of the trial context? | Yes – 3/22 ECD patients converted to PP intra-operatively (tumor proximity to facial nerve); per-protocol analysis used | Not explicitly reported; all patients appear to have completed assigned intervention |
| **2.3 Analysis approach** | Were participants analyzed in the group to which they were randomized (intention-to-treat)? | No – Protocol-based analysis; 3 converted patients moved to PP group, reducing ECD from 22 to 19 | ITT not formally stated; no reported conversions; likely analyzed as allocated |
|  | **Domain 2 Verdict →** | **Some concerns** | **Low** |
| **Domain 3: Bias in measurement of the outcome** | | | |
| **3.1 Outcome assessor blinding** | Were outcome assessors blinded to intervention assignment? | No – Same surgeons performed and followed up all cases; facial nerve assessment done by operating team | No – Same surgical team performed operations and follow-up; no independent assessor mentioned |
| **3.2 Outcome measurement** | Was the outcome measurement method likely to have introduced measurement bias? | Partially – Facial nerve grading criteria defined (8-week threshold for temporary vs. permanent); Frey's syndrome clinically defined. Histopathology independently confirmed. | Facial nerve outcomes not graded with validated scale (e.g., House-Brackmann); clinical assessment by treating team. Histopathology independently confirmed. |
|  | **Domain 3 Verdict →** | **Some concerns** | **Some concerns** |
| **Domain 4: Bias due to missing outcome data** | | | |
| **4.1 Missing outcome data** | Were data for this outcome available for all, or nearly all, randomized participants? | Apparently complete; all 46 patients (final allocation 27 PP, 19 ECD) reported at 12-month follow-up | Complete; all 50 patients followed up (mean 33–34 months); no withdrawals or losses reported |
| **4.2 Reasons for missing data** | If data were not available for all participants, is it likely that results were biased due to missing data? | N/A – No missing data reported | N/A – No missing data reported |
|  | **Domain 4 Verdict →** | **Low** | **Low** |
| **Domain 5: Bias in selection of the reported result** | | | |
| **5.1 Pre-registration / protocol** | Was the trial pre-registered with a prospectively specified analysis plan? | No trial registration number reported; no published protocol identified | No trial registration number reported; no published protocol identified |
| **5.2 Selective outcome reporting** | Were all pre-specified outcomes reported, or is there evidence of selective reporting? | Outcomes (facial nerve injury, Frey's syndrome, fistula, recurrence, return to work) appear complete but no protocol to verify against | Key outcomes (facial nerve injury, Frey's syndrome, fistula, recurrence, operative time) reported; no protocol available for verification |
|  | **Domain 5 Verdict →** | **Some concerns** | **Some concerns** |
| **OVERALL RISK OF BIAS** | | **Some concerns** | **Some concerns** |

ECD, extracapsular dissection; ITT, intention-to-treat; RoB 2, Cochrane Risk of Bias tool version 2; PP, partial parotidectomy.

Judgement categories: Low risk of bias | Some concerns | High risk of bias (per RoB 2 algorithm, as described in Sterne JAC, Savović J, Page MJ, Elbers RG, Blencowe NS, Boutron I, Cates CJ, Cheng HY, Corbett MS, Eldridge SM, Emberson JR, Hernán MA, Hopewell S, Hróbjartsson A, Junqueira DR, Jüni P, Kirkham JJ, Lasserson T, Li T, McAleenan A, Reeves BC, Shepperd S, Shrier I, Stewart LA, Tilling K, White IR, Whiting PF, Higgins JPT. RoB 2: a revised tool for assessing risk of bias in randomised trials. BMJ. 2019 Aug 28;366:l4898. doi: 10.1136/bmj.l4898. PMID: 31462531.).

Reference 46 in the main text:

Abdwahed M, El Azazy M, Mohsen SM. Short-term outcomes of extracapsular dissection versus superficial parotidectomy in the management of benign parotid tumors: A prospective comparative study. The Egyptian Journal of Surgery. 2022;41(3):1155-60. doi: 10.4103/ejs.ejs_209_22

Reference 60 in the main text:

Rafi Y, Sohail R, Balouch SS, Naqi SA. Superficial Parotidectomy and Extracapsular Dissection as Part of Surgeon's Armamentarium for Benign Parotid Tumors: A Clinical Trial. Annals of King Edward Medical University. 2020;26(3):456-61. doi: 10.21649/akemu.v26i3.4142

**Supplementary Table S5**

| **Supplementary Table S5.** These statements of the RoB-NMA tool [^36^](#_ENREF_36) were denied. |
| --- |
| 1. All interventions included in the network meta-analysis and their comparators are reasonable alternatives for the entire target population. This was denied because not every tumor can be treated with ECD. However, selected tumors for ECD can also be treated with PP. |
| 1. The participant characteristics influencing the effect were similar in the direct comparisons in the network. This was not always the case, as most studies were not randomized. As a result, small, easily removable tumors were sometimes compared with large tumors close to nerves, the removal of which can be associated with increased complications. |
| 1. The bias in the primary studies was minimal or was taken into account in the synthesis. Bias in the primary studies was not determined. |

**Supplementary Table S6**

| **Supplementary Table S6**. Overall assessment of bias according to ROBIS [^37^](#_ENREF_37) and ROB-NMA [^36^](#_ENREF_36), | | | | | | | | |
| --- | --- | --- | --- | --- | --- | --- | --- | --- |
| Network-  meta-analysis | ROBIS-Domains | | | ROB-NMA-Domains | | | Overall judgement | |
|  | Study eligibility criteria | Identification and selection of studies | Data collection and study appraisal | Interventions and network geometry | Effect  modifiers | Statistical synthesis | Results | Conclusions |
| Recurrence | **●** | **●** | **●** | **●** | **●** | **●** | **●** | **●** |

**● =** low risk of bias**, ● =** some risk of bias**, ● =** high risk of bias^,

**Supplementary Table S7**

| **Supplementary Table S7.** Previous meta-analysis. | | | | | | | | | |
| --- | --- | --- | --- | --- | --- | --- | --- | --- | --- |
| **No.** | **Authors** | **Year** | **No. of studies / patients** | **Patients’**  **histologies** | **Inter-**  **ventions** | **TRR** | **FPR** | **Outcome** | **Result** |
| 1 | Albergotti et al. [^15^](#_ENREF_15) | 2012 | 9 / 1,882 | Benign tumors | ECD, SP | ECD: 1.5%  SP: 2.4% | ECD: 1.4%  SP: 1.1% | TRR: ECD = SP  transient FPR: ECD < SP  permanent FPR: ECD = SP  Frey rate: ECD < SP | Follow-up:  0-32 years |
| 2 | Colella et al. [^21^](#_ENREF_21) | 2015 | 20 / 2674 | PA | ECD, PP. SP, TP, even enucleation | TP: 1%  SP: 2%  PP: 2%  ECD: 1%  Enucleation: 8% | Not reported | The different techniques were not compared with each other. | Follow-up:  5-11 years |
| 3 | Foresta et al. [^16^](#_ENREF_16) | 2014 | Variable 4-11 / unclear | Two analyses  PA alone and benign tumors | ECD, SP | *per 1000 persons-years  PA:  ECD: 1.4  sP: 3.0  All:  ECD: 0.3  SP: 5.3 | *per 1000 persons-years  PA:  ECD: 1.1  SP: 2.2  All:  ECD: 1.6  SP: 3.3 | ECD and SP separately analyzed; no comparison of ECD and SP was performed | Follow-up:  >5 years |
| 4 | Li et al. [^19^](#_ENREF_19) | 2020 | 11 / 1,272 | Benign tumors | Two different PP techniques: SP and PSP | PSP: 1.0%  SP: 0.5% | PSP: 1.2%  SP: 2.5% | TRR: PSP = SP  transient FPR: PSP < SP  transient or  permanent FPR: PSP = SP  Frey rate: PSP < SP | Range:  6 months to 8 years |
| 5 | Lin et al. [^18^](#_ENREF_18) | 2019 | 7 / 1,641 | Benign tumors | ECD, PSP | ECD: 0.0%  PSP: 1.5% | ECD: 1.8%  PSP: 2.5% | TRR: ECD = PSP  transient FPR: ECD < PSP  permanent FPR: ECD = PSP  Frey rate: ECD = PSP  Infection: ECD = PSP | Mean:  12 - 28 months |
| 6 | Liu et al. [^20^](#_ENREF_20) | 2023 | 23 / 2,844 | Benign tumors | Two different PP techniques: SP and PSP | PSP: 1.8%  SP: 1.4% | PSP: 1.0%  SP: 3.3% | TRR: PSP = SP  transient FPR: PSP < SP  permanent FPR: PSP < SP  Frey rate: PSP < SP | Range:  6 - 62 months |
| 7 | Mashrah et al. [^22^](#_ENREF_22) | 2021 | 44 / 7,841 | Benign tumors | Pairwise and network meta-analysis:  ENU; ECD, PSP, SP, TP | ENU: 14.3%  ECD: 3.6%  PSP: 3.7%  SP: 2.8%  TP: 1.4% | ENU: 0.0%  ECD: 1.2%  PSP: 1.3%  SP: 2.7%  TP: 2.9% | TRR: ENU > ECD, SPS, SP, and TP  TRR: ECD = PSP = SP = TP  transient FPR: ECD < PSP  PSP < SP  permanent FPR: ECD < PSP  PSP < SP  SP < TP | 12 months - 14 years |
| 8 | Xie et al. [^17^](#_ENREF_17) | 2015 | 14 / 3,194 | Benign tumors | ECD, SP | ECD: 1.5%  SP: 1.4% | ECD: 1.1%  SP: 2.0% | TRR: ECD = SP  transient FPR: ECD < SP  permanent FPR: ECD < SP  Frey rate: ECD < SP | 0 - 30 years |

Enucleation; ECD = extracapsular dissection; PP = partial parotidectomy; TP = total parotidectomy; SP = superficial parotidectomy; PSP = partial superficial parotidectomy; TRR = tumor recurrence rate; FPR = permanent facial palsy rate; PA = pleomorphic adenoma; WT = Warthin tumor; Frey= Frey syndrome. *not the absolute rate was calculated, but the number of cases per 1000 persons-years.

**Supplementary Figure S1**


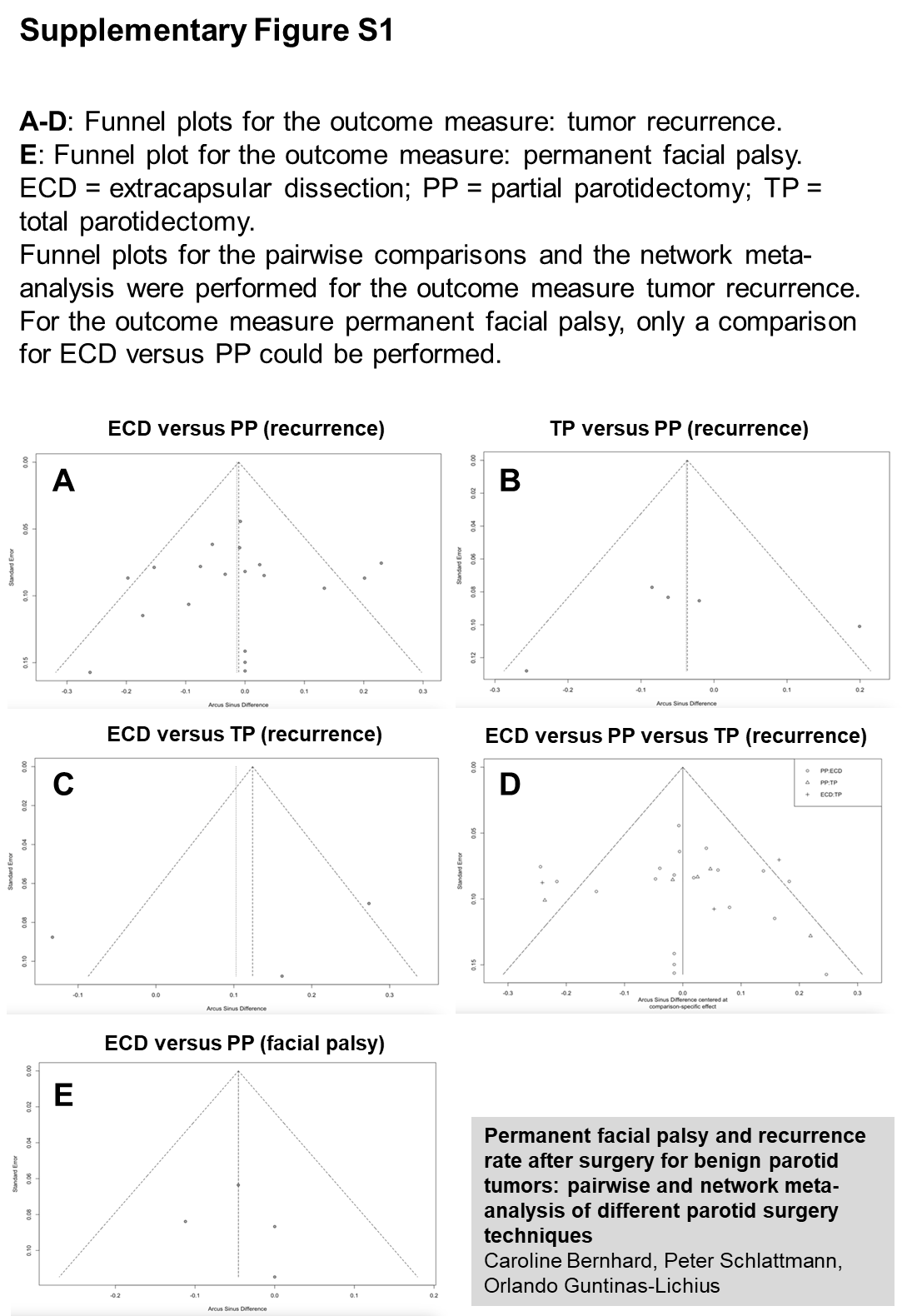

Supplement: Supplementary file 1 [file Supplementaryfile1.docx]
